# An annotated checklist of the vascular flora of South and North Nandi Forests, Kenya

**DOI:** 10.3897/phytokeys.155.51966

**Published:** 2020-08-07

**Authors:** David Kimutai Melly, Solomon Kipkoech, Benjamin Watuma Muema, Peris Kamau, Itambo Malombe, Guangwan Hu, Qing-Feng Wang

**Affiliations:** 1 CAS Key Laboratory of Plant Germplasm Enhancement and Specialty Agriculture, Wuhan Botanical Garden, Chinese Academy of Sciences, Wuhan 430074, Hubei, China; 2 University of Chinese Academy of Sciences. Beijing 100049, China; 3 Sino-Africa Joint Research Center, Chinese Academy of Sciences, Wuhan 430074, Hubei, China; 4 East African Herbarium, National Museums of Kenya, P.O. Box 45166 00100, Nairobi, Kenya

**Keywords:** Biodiversity, conservation, floristic survey, inventory, Nandi County

## Abstract

We compiled a checklist of the flora of South and North Nandi forests based on literature, online databases, herbarium collections and floristic field surveys. A combination of general walk-over surveys and plotless landscape sampling for plant collection and sight observation was used. We recorded 628 plant species representing 118 families and 392 genera, which almost double the latest results of the previous most recent survey. We found 61 species of ferns and fern allies and 567 species of seed plants, representing 9.98% of the total plant species in Kenya. Herbs were the majority (50.2%) of life forms followed by shrubs (16.5%). We report unique populations of three species out of 19 species that are widespread in Africa, but restricted to Nandi and Kakamega Forests in Kenya. Four of the recorded species are threatened globally and 16 exotic plant species were found. The recent description of one new species and two new records for Kenya from these forests, together with the comprehensive checklist is of crucial importance to the conservation of these unique ecosystems. Our results are essential to forest managers, community forest associations, conservationists, students and research scientists in Kenya and globally for implementing critical decisions for the conservation of this vital biodiversity resource.

## Introduction

Tropical rainforests support more than half of the world’s species (Armstrong 2016; McFarland 2018), although they constitute only 7% of Earth’s land area. Nevertheless, no other land community sustains such a high species diversity and ecological complexity like tropical rainforests (Lewis 2006). As a result, they are very critical and essential in biodiversity conservation. Tropical forest ecosystems are crucial, because they act as reservoirs of biodiversity, they are sources of timber and medicinal plants, they also act as carbon sinks and play a critical role in watershed protection (Russo and Kitajima 2016; Maua et al. 2018a). Due to their diversity, tropical rainforests provide habitat for more than half of the world’s known terrestrial plant and animal species (Lewis 2006; McFarland 2018). Globally, most forests experience enormous fragmentation as the human population increases; hence more land is needed to cater for human needs (Mitchell et al. 2006). Tropical forests are at the forefront of species extinction crises due to widespread habitat loss and alteration (Mwavu and Witkowski 2009; Vuyiya et al. 2014; Tanui 2015; Mutoko et al. 2015). Between 2000 and 2012, “the world has been losing about 0.43% of its remaining tropical rainforests per year (Lewis 2006; McFarland 2018). Moreover, it is estimated that only 2.5% of the surface area of Earth is covered by rainforests, or approximately 8% of the land on Earth consists of rainforests (Pariona 2018). If this rate of loss persists, one-third of all remaining tropical rainforests will be primarily altered in the next 30 years (Althof 2005). Forests like North Nandi and South Nandi are diminishing due to the increased demand for their useful products and services (Mutoko et al. 2015). The tropical rainforests have received increasing attention both globally and locally due to their importance, with numerous efforts and calls for its proper assessment, management, conservation and documentation of their biodiversity status in a bid to prevent them from being wiped out.

Kenya has the most diverse forests in East Africa (Peltorinne 2004). They harbour over 6,000 species of higher plants, including 2,000 trees and shrubs (KIFICON 1994a; MEWNR 2015; Zhou et al. 2017). Although they are highly fragmented, these forests are biologically rich and harbour high concentrations of endemic species of animals and plants (Peltorinne 2004).

The Nandi Forests are within Nandi County in Western Kenya and occupy an area currently covered by three sub-counties, namely; Nandi Central, Nandi South and Kabiyet (Ehrich et al. 2007). They are enriched by Afromontane forest elements from the Rift Valley escarpment (Fischer et al. 2010). These two forests have been classified as semi-humid and as a nature reserve (Kigomo 1987). In the years from the 1980s and earlier, Kenya’s indigenous forest coverage was about 2% (Wass 1995) and agricultural communities occupied most of the remaining areas. Some parts of the Nandi forests constitute a lowland rainforest-like Kakamega Tropical Rainforest (MEWNR 2015).

A careful review of the literature available for North Nandi and South Nandi Forests indicates that only a few floristic studies have been done in the past. Most plant species have not been fully documented and, if there is any documentation, it focuses on tree species (Koros et al. 2016). Previous research on these forests includes ethnobotany studies, which recorded 56 plant species in 30 families that are useful in treating reproductive disorders (Pascaline et al. 2011). The list of tree species in North Nandi forest, used in the study by Diamond and Fayad (1979) while undertaking their Avifauna studies, had 56 species. An inventory by KIFCON (1994a, b), that concentrated on the total standing volume of tree species, recorded 79 species in South Nandi and 65 species in North Nandi. A team from the National Museums of Kenya, who surveyed the biodiversity of these forests, recorded 125 plant species in South Nandi and 171 plant species in North Nandi (Musila et al. 2011). Finally, Maua et al. (2018a) found 128 plant species belonging to 105 genera and 55 families that are used as Non-Timber Forest Products (NTFPs) by households adjacent to South Nandi Forest.

The socioeconomic factors influencing the dependence of households on non-timber forest products in South Nandi Forest were addressed by Maua et al. (2018b). The most recent study about the floristic structure and plant composition in Nandi Forests (Girma et al. 2015) found 321 plant species in 92 families and 243 genera.

Other studies focused on the ecology, species distribution and composition, as well as the management of these forests by different stakeholders (Njunge and Mugo 2011; Mbuvi et al. 2015; Tanui 2015; Koros et al. 2016).

This study fills the existing knowledge gap by reporting on the indigenous flora of Nandi forests located near the remaining Kakamega Rainforest in Kenya. Our aim is to provide the first comprehensive, detailed checklist of the flora situated in this important biodiversity area to help in conservation and management, as well as determine the plant species composition and structure of North and South Nandi Forests of Kenya.

## Material and methods

### Study site and current vegetation status

**Figure 1.**
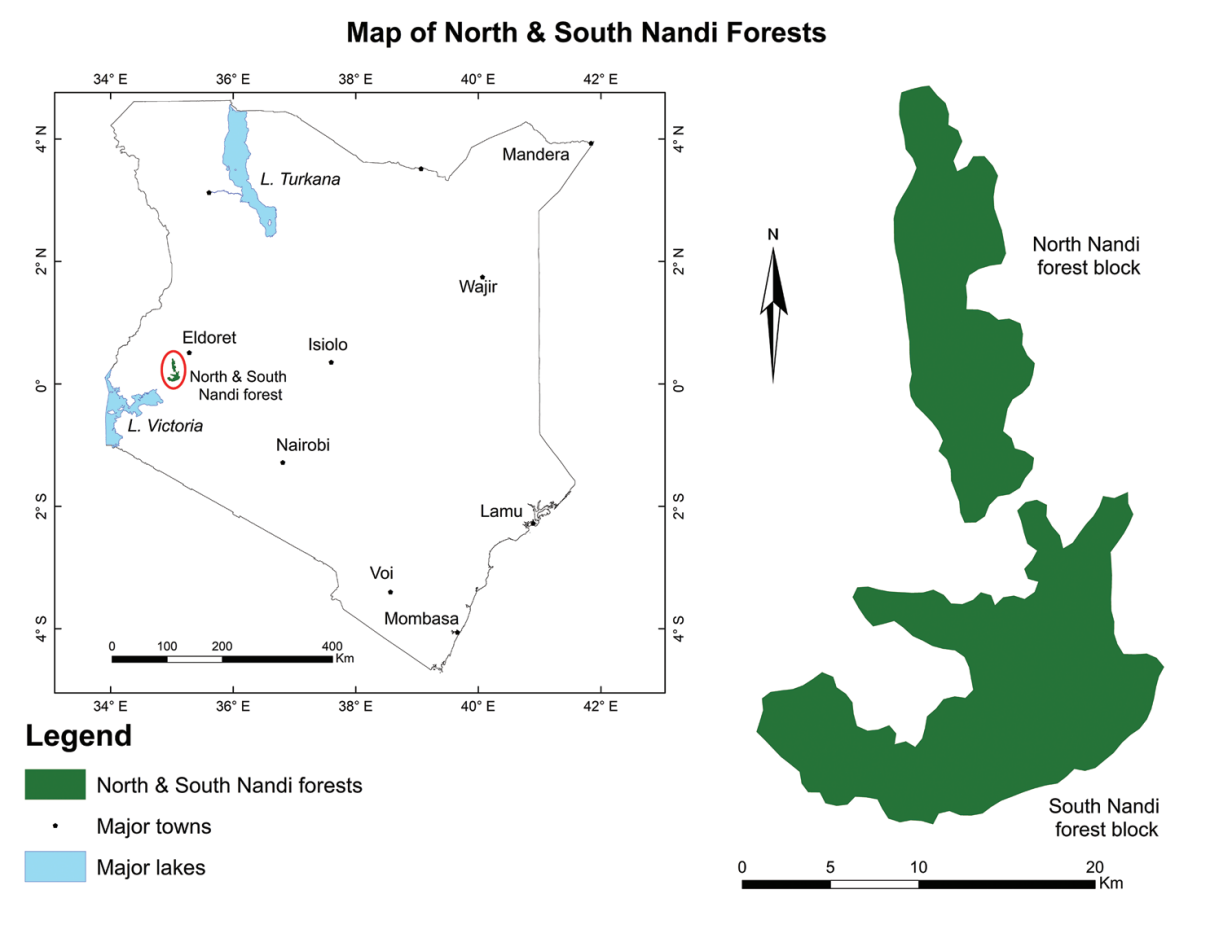
Location of North and South Nandi forests of Kenya.

Nandi Forests (Fig. 1) are situated on the top of Nandi escarpment in the Rift Valley Province of Kenya to the east of Kakamega Forest (KIFCON 1994a; 1994b). It comprises of two forests; North Nandi to the north of Kapsabet town and measures about 10,501 ha (KIFCON 1994b) and it stretches for more than 30 km from north to south. It is seldom more than 5 km wide or less than 3 km wide for a considerable part of its length (KIFCON 1994b) while South Nandi Forest is situated to the south of North Nandi Forest and covers 19,502 ha (KIFCON 1994a). North Nandi Forest, together with the Kakamega Forest and South Nandi Forest, is one of the three forests in western Kenya, southeast of Mount Elgon (Schifter et al. 1998). North Nandi forest is found in Nandi North District between 00°12.38'–00°25.10'N latitude and 34°57.58'–35°01.05'E longitude. South Nandi Forest is situated in Nandi South District between latitude 00°34'N and 35°25'E (Maua et al. 2018b). The elevation of Nandi Forests is between 1695 and 2145 m. The mean annual rainfall is 1800 to 2000 mm, with peaks in April/May and August/September. (Mitchell et al. 2006; Lung and Schaab 2010; Maua et al. 2018b).

The mean annual temperature ranges between 17 °C and 20 °C, with the mean maximum and minimum of 25 °C and 11 °C, respectively (KIFCON 1994a). The soils of the North Nandi Forest are derived from undifferentiated basement system rocks and are well-drained, deep and red to yellowish-red friable sandy clays (KIFCON 1994a). The most common tree species communities of Nandi Forests are: *Diospyros abyssinica*-*Heinsenia diervilleoides*, *Trilepisium madagascariense*-*Solanum mauritianum* and *Turraea holstii*-*Ehretia cymose*. Some of the dominant vascular plant species present includes *Croton macrostachyus*, *Celtis africana*, *Strombosia scheffleri*, *Syzygium guineense*, *Tabernaemontana stapfiana*, *Casearia battiscombei*, *Croton magalocarpus*, *Macaranga capensis* and *Neoboutonia macrocalyx* (Girma et al. 2015; KIFCON 1994a).

### Floristic surveys, specimen collection and identification

Floristic surveys and specimen collections were conducted between November 2016 and April 2019. The botanical team consisted of botanists from the National Museums of Kenya and the Sino-Africa Joint Research Center (SAJOREC). Our study covered the entire forest from 40 sites within the two forests, 20 in South Nandi and 20 in North Nandi Forest. A combination of general walk-over survey method (Filgueiras et al. 1994) and a plotless landscape defined sampling methods for plant specimen collection and sight observation was used to aid the characterisation of the vegetation (Hall and Swaine 1981). Plant specimens bearing flowers or fruit were collected and identified. The habit, habitat, elevation and collector details were recorded. The samples were then preserved by pressing. All the plant specimens collected are stored at the East Africa Herbarium (EA) and Wuhan Botanical Garden (HIB).

All vascular plant specimens, that were previously collected from the entire former Nandi District and particularly North and South Nandi Forests, were compiled from 43 herbarium specimens in the East Africa Herbarium at the National Museums of Kenya. Additionally, the species collected in the previous decades are reported in this checklist. These were obtained by searching the locality “Nandi District” records in literature (FTEA 1952–2012, Beentje et al. 1994, Agnew and Agnew 1994; Agnew 2013). These botanical references were also used to identify the specimens that we collected. The state of endemism was evaluated by searching all the vascular plants recorded, including existing endemics cited in literature in the Global Biodiversity Information Facility (GBIF) (www.gbif.org). The conservation status of all the vascular plant species collected was assessed using the criteria from the International Union for Conservation of Nature (IUCN 2019) (https://www.iucnredlist.org/).

Habitat description for each species was based on our field observations. For those species that we collected more than one specimen, maximum and minimum altitude ranges are recorded in the checklist. For the herbarium specimens, their habitats were not restricted to the Nandi Forests, since some of the species had wide distribution ranges. The life form information was obtained from field observations and botanical literature. They were categorised as trees (main trunk over 3 m tall, shrubs (0.5–3 m plants with woody stems branching near the ground), climbers (with twinning herbaceous or woody stems) and herbs (< 0.5 m, or < 1 m without persistent woody stems).

Plant taxonomic circumscription and authorities for each taxon were checked in the African Plant Database (https://www.ville-ge.ch/musinfo/bd/cjb/africa/index.php?langue=an) and Catalogue of Life, 2019 Annual Checklist (http://www.catalogueoflife.org/) and further verified using the International Plant Names Index’ (). Herbarium acronyms followed Thiers (2016).

### Checklist

A comprehensive checklist of the vascular plant taxa of the Nandi Forests is enumerated below. Families are grouped in lycophytes, monilophytes, gymnosperms and angiosperms. Families in lycophytes and monilophytes follow the PPG I system (PPG I 2016), those in gymnosperms are based on Christenhusz et al. (2011) and families in angiosperms are based on the APG IV system (Chase et al. 2016). Recently-described species and new floristic records for Kenya are indicated in the checklist. Asterisk (*) before the name indicates the introduced species. The conservation status of vulnerable taxa is shown at the end of the taxon.

## Results and discussion

### Families, genera and species diversity

The current list contains 628 vascular plant taxa representing 118 families and 392 genera. Out of the total species recorded, 43 species were obtained from herbarium specimens at the National Museums of Kenya. Lycophytes and monilophytes comprised 61 species. Seed plants included 567 species, representing 9.98% of the 6,293 total vascular plant species in Kenya recorded by the Flora of Tropical East Africa. Angiosperms represent 90.12% of the total species collected in Nandi Forests (357 genera, 566 species), followed by monilophytes with 9.4% (33 genera, 59 species), lycophytes with 0.3% (1 genus, 2 species) and gymnosperms with 0.2% (1 genus, 1 species).

The top five species-rich families of vascular plants in this checklist were Asteraceae, Orchidaceae, Fabaceae, Poaceae and Lamiaceae (Table 1). The top five species-rich genera were *Asplenium* (Aspleniaceae), *Cyperus* (Cyperaceae), *Polystachya* (Orchidaceae), *Solanum* (Solanaceae), *Ficus* (Moraceae), *Impatiens* (Balsaminaceae) and *Plectranthus* (Lamiaceae) (Table 1).

**Table 1.** Top 10 species-rich families and genera of North and South Nandi forest.

Top 10 Genera			Top 10 Species-rich families		
Family	Genus	Species	Family	Genera	Species
Aspleniaceae	* Asplenium *	20	Asteraceae	36	50
Cyperaceae	* Cyperus *	10	Orchidaceae	17	37
Orchidaceae	* Polystachya *	8	Fabaceae	21	31
Solanaceae	* Solanum *	8	Poaceae	19	30
Moraceae	* Ficus *	7	Lamiaceae	14	28
Balsaminaceae	* Impatiens *	7	Cyperaceae	9	26
Lamiaceae	* Plectranthus *	7	Rubiaceae	18	23
Orchidaceae	* Bulbophyllum *	6	Aspleniaceae	1	20
Asparagaceae	* Chlorophytum *	5	Acanthaceae	10	18
Poaceae	* Eragrostis *	5	Euphorbiaceae	10	14

### Plants life forms

The majority of the plants were herbs comprising 50.2% of the total plant species, followed by shrubs, trees, climbers, epiphytes, shrubs or small trees, lianas and subshrubs (Table 2).

**Table 2.** Life forms of plants species of North and South Nandi forests.

Life form	Species	Percentage of species recorded (%)
Herbs	316	50.2
Shrubs	104	16.5
Trees	81	12.9
Climbers	59	9.4
Epiphytes	34	5.4
Shrub or small trees	28	4.4
Lianas	6	0.9
Subshrubs	2	0.3

### Plant species of special concern

**Exotic species**

We recorded 16 introduced or exotic plant species (3.02%) belonging to 13 genera and eight families (Table 3). These plants represented 2.5% of the total plants collected in Nandi Forests. The families with more species were Fabaceae (4), Asteraceae (3) and Solanaceae (3). All these plants originated from eight different regions (Table 3). Most of the introduced species were from South America (7), Central and South America (2) (Table 3).

**Table 3.** List of exotic plant species in North and South Nandi Forests.

Family	Species	Native/Origin
Apocynaceae	*Gomphocarpus physocarpus* E. Mey.	Southern Africa
Asteraceae	*Acanthospermum glabratum* (DC.) Wild	South America
Asteraceae	*Ageratum conyzoides* L.	South America
Asteraceae	*Tagetes minuta* L.	South America
Fabaceae	*Caesalpinia decapetala* (Roth) Alston	India & Southeast Asia
Fabaceae	*Senna didymobotrya* (Fresen.) H. S. Irwin & Barneby	Tropical Africa
Fabaceae	*Senna obtusifolia* (L.) H. S. Irwin & Barneby	Tropical America
Fabaceae	*Senna septemtrionalis* (Viv.) H. S. Irwin & Barneby	Mexico & Central America
Myrtaceae	*Eucalyptus saligna* Sm.	Australia
Passifloraceae	*Passiflora edulis* Sims	South America
Phytolaccaceae	*Phytolacca octandra* L.	South America
Solanaceae	*Cestrum aurantiacum* Lindl.	Tropical America from Guatemala to Venezuela
Solanaceae	*Physalis peruviana* L.	South America
Solanaceae	*Solanum mauritianum* Scop.	South America
Verbenaceae	*Lantana camara* L.	Central & South America
Verbenaceae	*Lantana trifolia* L.	Central & South America

**Widespread taxa restricted in Kenya to Nandi and Kakamega Forests**

The checklist contains 19 species that are widespread in Africa but are restricted in Kenya to the Kakamega and Nandi Forests (Table 4). Other than the species in Table 4, one species *Alchornea hirtella* Benth. is also widespread in Africa. Still, in Kenya, it is restricted to Kakamega, Nandi and the Coastal forests.

**Table 4.** Taxa restricted to Kakamega and Nandi forests in Kenya, but widespread in Africa.

Family	Species	Life form
Acanthaceae	*Pseuderanthemum ludovicianum* (Büttner) Lindau	Herb
Amaranthaceae	*Sericostachys scandens* Gilg & Lopr.	Shrub
Athyriaceae	*Diplazium velaminosum* (Diels) Pic. Serm	Herb
Celastraceae	*Salacia cerasifera* Wele. Ex Oliv.	Shrub
Celastraceae	*Simirestis brianii* N. Hallé	Shrub
Cucurbitaceae	*Coccinia barteri* (Hook. f.) Keay	Climber
Cucurbitaceae	*Coccinia subsessiliflora* Cogn.	Climber
Cucurbitaceae	*Momordica cissoides* Benth.	Climber
Cucurbitaceae	*Zehneria longiflora* G. W. Hu & Q. F. Wang	Climber
Lamiaceae	*Achyrospermum parviflorum* S. Moore	Herb
Lamiaceae	*Clerodendrum formicarum* Gűrke	Shrub
Lamiaceae	*Clerodendrum melanocrater* Gürke	Shrub
Orchidaceae	*Bulbophyllum encephalodes* Summerh.	Epiphyte
Orchidaceae	*Nervilia lilacea* Jum. & H. Perrier	Herb
Passifloraceae	*Adenia bequaertii* Robyns & Lawalrée	Climber
Passifloraceae	*Adenia cissampeloides* (Planch. ex Hook.) Harms	Climber
Proteacae	*Protea madiensis* Oliv.	Shrub
Sapotaceae	*Synsepalum cerasiferum* (Welw.) T. D. Penn.	Tree
Vitaceae	*Cissus humbertii* Robyns & Lawalrée	Climber

**Threatened species**

A total of four species; *Arachniodes webbiana* (A. Braun) Schelpe (Dryopteridaceae), *Agelanthus pennatulus* (Sprague) Polhill & Wiens (Loranthaceae), *Prunus africana* (Hook. f.) Kalkman (Rosaceae) and *Cissus humbertii* Robyns & Lawalrée (Vitaceae) were found to be vulnerable globally, 234 species were of least concern. In comparison, 392 vascular plant species in this checklist have not been evaluated (Source: IUCN 2019); these represent 0.6% of the total plant species recorded for North and South Nandi forests. The high percentage (62.22%) of the plants that have not been assessed in Nandi Forests call for the need for assessment to conserve those species that are of special concern.

**New floristic records**

During the fieldwork of December 2016, we found a new species of *Zehneria* (Wei et al. 2017) from South Nandi Forest. In subsequent field collections, 2017 and 2018, we collected the first record of *Coccinia subsessiliflora* Cogn. (Melly et al. 2019) for Kenya, a widespread species in tropical Africa. *Nervilia lilacea* Jum. & H. Perrier (Orchidaceae) (Jing Tian et al. 2019) is also a new record for Kenya and is the only record for the northern hemisphere.

### Checklist

Besides the taxon name, each record includes information about habit, habitat, elevation (m), representative herbarium vouchers. Vulnerable species are labeled as such. Herbaria: EA (East African Herbarium, Kenya), HIB (Herbarium of Wuhan Botanical Garden, China). Collecting teams: FOKP (Flora of Kenya Project) and Sino Africa Joint Investigation Team (SAJIT).

LYCOPHYTES

**F1. Lycopodiaceae**

1 Genus, 2 species

***Huperzia dacrydioides* (Baker) Pic. Serm.** Epiphyte. Upland forest, 1928–2364 m. FOKP 1468 (EA, HIB), FOKP 1494 (EA, HIB).

***Huperzia gnidioides* (L. f.) Trevis.** Epiphyte. Wet upland forest, 1925 m. FOKP 1461 (EA, HIB).

MONILOPHYTES

**F2. Aspleniaceae**

1 Genus, 20 species

***Asplenium abyssinicum*** Fée. Herb. Under upland forest, 2342 m. FOKP 1493 (EA, HIB).

***Asplenium aethiopicum* (Burm. f.) Bech.** Perennial herb. Dry and moist forest, moist bushland on tree, 1872–2052 m. FOKP 1318 (EA, HIB), FOKP 1440 (EA, HIB), SAJIT 006956, (EA, HIB).

***Asplenium boltonii* Hook. ex Schelpe.** Perennial herb. Dry and moist forest, moist bushland on tree, 2052 m. FOKP 1362 (EA, HIB).

***Asplenium bugoiense* Hieron.** Herb. Moist upland forest, 1989 m. FOKP 1366 (EA, HIB), FOKP 1402 (EA, HIB).

***Asplenium ceii* Pic. Serm.** Herb. Dense shade in moist forests, 1974–1989 m. FOKP 1393 (EA, HIB).

***Asplenium elliottii* C. H. Wright.** Perennial herb. Dense shade in moist forests, 1975–2050 m. FOKP 1359 (EA, HIB), FOKP 1490 (EA, HIB).

***Asplenium erectum* Bory ex Willd.** Herb. Dense shade, seasonally moist forest, 1971–2125 m. FOKP 1387 (EA, HIB), FOKP 1363 (EA, HIB).

***Asplenium friesiorum* C. Chr.** Herb. Moist upland forest, 1925 m. FOKP 1463 (EA, HIB).

***Asplenium gemmiferum* Schrad.** Perennial herb. Dense shade of Moist forests, 1810–2397 m. FOKP 1492 (EA, HIB), SAJIT 006962 (EA, HIB), Melly 0305 (EA).

***Asplenium hypomelas* Kuhn.** Herb. In moist montane forests, generally along the rivers, 1927–2007 m. FOKP 1426 (EA, HIB), FOKP 1443 (EA, HIB).

***Asplenium lividum* Mett. ex Kuhn.** Perennial herb. Moist forest, 1000–2550 m. Gillet 16698 (EA).

***Asplenium macrophlebium* Baker.** Herb. Moist forest, 1000–1850 m. Faden and Rathbun 69/2118 (EA).

***Asplenium mannii* Hook.** Epiphytic herb. Moist intermediate and montane forest, 2036–2087 m. FOKP 1508 (EA, HIB).

***Asplenium megalura* Hieron.** Perennial herb. moist intermediate and montane forests, 1893–1969 m. FOKP 1437 (EA, HIB), SAJIT 006942 (EA, HIB).

***Asplenium protensum* Schrad.** Herb. Moist upland forest, 1975 m. FOKP 1365 (EA, HIB), FOKP 1368 (EA, HIB), FOKP 1434 (EA, HIB).

***Asplenium sandersonii* Hook.** Herb. Montane moist forest, occasionally in moister spots in dry forests, 1975 m. FOKP 1665 (EA, HIB).

***Asplenium smedsii* Pic. Serm.** Epiphyte. Moist montane forest, 2010–2257 m. FOKP 1401 (EA, HIB) FOKP 1433 (EA, HIB), FOKP 1472 (EA, HIB).

***Asplenium stuhlmannii* Hieron.** Epiphyte. Moist montane forest, 2010 m. FOKP 1533 (EA, HIB), FOKP 1608 (EA, HIB).

***Asplenium theciferum* (Kunth) Mett.** Perennial herb. Moist upland forest, 1920 m. FOKP 1319 (EA, HIB).

***Asplenium thunbergii* Kunze.** Herb. Dense shade of Moist forests, 1961 m. FOKP 1412 (EA, HIB).

**F3. Athyriaceae**

3 Genera, 3 species

***Athyrium scandicinum* (Willd.) C. Presl.** Herb. Montane forests extending along the streams, 2016–2063 m. FOKP 1521 (EA, HIB), FOKP 1681 (EA, HIB).

***Deparia boryana* (Willd.) M. Kato.** Herb. Moist upland forest, 1966–2007 m. FOKP 1424 (EA, HIB).

***Diplazium velaminosum* (Diels) Pic. Serm.** Herb. Along streams in upland evergreen forest, 1927 m. FOKP 1454 (EA, HIB).

**F4. Blechnaceae**

1 Genus, 1 species

***Blechnum attenuatum* (Sw.) Mett.** Herb. Moist montane forest, 1929 m. FOKP 1471 (EA, HIB).

**F5. Cyatheaceae**

1 Genus, 1 species

***Alsophila manniana* (Hook.) R. M. Tryon.** Tree. Form dense stand in steep, forested valleys along rivers, roadsides, 1991 m. Mabberley 19 (EA).

**F6. Cystopteridaceae**

1 Genus, 1 species

***Cystopteris fragilis* (L.) Bernh.** Herb. Upland forest, 2016 m. FOKP 1520 (EA, HIB).

**F7. Dennstaedtiaceae**

1 Genus, 1 species

***Hypolepis sparsisora* (Schrad.) Kuhn. Herb.** Common in open areas at high altitudes, 1996 m. FOKP 1562 (EA, HIB).

**F8. Didymochlaenaceae**

1 Genus, 1 species

***Didymochlaena truncatula* (Sw.) J. Sm.** Herb. Wet forests, 2019 m. FOKP 1403 (EA, HIB), FOKP 1404 (EA, HIB).

**F9. Dryopteridaceae**

4 Genera, 6 species

***Arachniodes webbiana* (A. Braun) Schelpe**. Herb. Wet upland forests, 2393 m. FOKP 1491 (EA, HIB). Vulnerable.

***Ctenitis cirrhosa* (Schumach.) Ching**. Herb. Wet upland forests, 2005–2193 m. FOKP 1399 (EA, HIB), FOKP 1442 (EA, HIB), FOKP 1516 (EA, HIB).

***Dryopteris pentheri* (Krasser) C. Chr.** Herb. Shades of moist forests and roadside banks in ditches, 1993 m. FOKP 1416 (EA, HIB).

***Dryopteris schimperiana* (Hochst.) C. Chr.** Herb. Riverine forest, 2008 m. FOKP 1417 (EA).

***Dryopteris manniana* (Hook.) C. Chr.** Herb. Moist upland forest, 1995 m. FOKP 1428 (EA).

***Polystichum sinense* (Christ) Christ.** Herb. Shady moist forest, along the streams, 2391 m. FOKP 1473 (EA, HIB).

**F10. Hymenophyllaceae**

1 Genus, 2 species

***Crepidomanes chevalieri* (Christ) Ebihara & Dubuisson.** Herb. Found on tree trunks in upland forest, 2036 m. FOKP 1509 (EA, HIB).

***Crepidomanes melanotrichum* (Schltdl.) J. P. Roux.** Herb. Usually on tree trunks, often in wet places in dry forests, 2036–2387 m. FOKP 1476 (EA, HIB), FOKP 1510 (EA, HIB).

**F11. Lomariopsidaceae**

1 Genus, 1 species

***Nephrolepis undulata* (Afzel. ex Sw.) J. Sm.** Herb. Moist thickets, 2041 m. FOKP 1635 (EA, HIB).

**F12. Marattiaceae**

1 Genus, 1species

***Ptisana fraxinea* (Sm.) Murdock.** Herb. Upland forest, 1989 m. FOKP 1370 (EA, HIB).

**F13. Polypodiaceae**

4 ***Genera***, 4 species

***Lepisorus excavatus* (Bory ex Willd.) Ching.** Epiphyte. Upland forest, moist, riverine forest, 1849–2140 m. FOKP 1358 (EA, HIB), FOKP 1639 (EA, HIB), FOKP 1648 (EA, HIB).

***Loxogramme abyssinica* (Baker) M. G. Price.** Herb. Dry and wet upland forest, 1873–1895m. FOKP 1351 (EA, HIB), SAJIT 006941 (EA, HIB).

***Pleopeltis macrocarpa* (Bory ex Willd.) Kaulf.** Herb. Dry or moist, riverine forest, 1893–2040 m. FOKP 1361 (EA, HIB), SAJIT 006940 (EA, HIB).

***Hovenkampia schimperiana* (Mett. ex Kuhn) Li Bing Zhang & X. M. Zhou.** Herb. Locally common on rocks and trees in riverine and moist intermediate forests, 600–2125 m. FOKP 1618 (EA, HIB).

**F14. Pteridaceae**

7 Genera, 10 species

***Aleuritopteris farinosa* (Forsk.) Fée.** Herb. Upland forest, 2180 m. FOKP 1505 (EA, HIB).

***Cheilanthes bergiana* Schltdl.** Herb. Moist banks in upland forest, 1966 m. FOKP 1397 (EA, HIB), FOKP 1398 (EA, HIB), FOKP 1522 (EA, HIB).

***Cheilanthes viridis* (Forssk.) Sw.** Herb. On banks, path sides, forest margins, among rocks in woodland, 1982 m. FOKP 1553 (EA, HIB).

***Coniogramme africana* Hieron.** Herb. Moist forest, 2013 m. FOKP 1408 (EA, HIB).

***Doryopteris concolor* (Langsd. & Fisch.) Kuhn.** Herb. Dry and wet forest, 2043 m. FOKP 1378 (EA, HIB), SAJIT 007022 (EA, HIB).

***Doryopteris kirkii* (Hook.) Alston.** Herb. Dry and wet forest, 2009 m. FOKP 1375 (EA, HIB).

***Haplopteris volkensii* (Hieron.) E. H. Crane.** Herb. Moist upland forest, 2016 m. FOKP 1519 (EA, HIB).

***Pellaea calomelanos* (Sw.) Link.** Herb. On the rock crevices or on roadside banks I full sun, 2125–2148 m. FOKP 1606 (EA, HIB), SAJIT 006611 (EA, HIB).

***Pteris dentata* Forssk.** Herb. Upland forest, moist areas, 1996–2019 m. FOKP 1391 (EA, HIB), FOKP 1563 (EA, HIB), FOKP 1627 (EA, HIB).

***Pteris pteridioides* (Hook.) Ballard.** Herb. Upland forest, moist areas, 1996–2019 m. FOKP 1388 (EA, HIB).

**F15. Tectariaceae**

2 Genera, 2 species

***Arthropteris orientalis* (J. F. Gmel.) Posth.** Herb. Partial shade on rocks or ground, rarely epiphytic, in dry or moist forests or thickets, 2140 m. FOKP 1336 (EA, HIB), Sangai573 (EA).

***Tectaria gemmifera* (Fée) Alston.** Herb. Upland forest, 1864 m. Melly 0247 (EA).

**F16. Thelypteridaceae**

4 Genera, 5 species

***Christella dentata* (Forssk.) Brownsey & Jermy.** Herb. Common along streams and other wet places in the forests, 1864 m. FOKP 1326 (EA, HIB).

***Cyclosorus gueinziana* (Mett.) J. P. Roux.** Herb. Riverine, moist roadside banks, 1864 m. FOKP 1332 (EA, HIB).

***Cyclosorus interruptus* (Willd.) H. Itô.** Herb. Marshes and swamps, 1864–1995 m. FOKP 1327 (EA, HIB), FOKP 1438 (EA, HIB), FOKP 1586 (EA, HIB).

***Pneumatopteris unita* (Kunze) Holttum.** Herb. Along streams in moist forest, 1771 m. Melly 0277 (EA).

***Pseudocyclosorus pulcher* (Bory ex Willd.) Holttum.** Herb. Shaded moist forest floors and next to streams, 1927 m. FOKP 1449 (EA, HIB).

GYMNOSPERMS

**F17. Podocarpaceae**

1 Genus, 1 species

***Podocarpus latifolius* (Thunb.) R. Br. ex Mirb.** Tree. Upland rainforest, 900–3150 m. Geesteranus 4984 (EA).

ANGIOSPERMS

**F18. Achariaceae**

1 Genera, 1 species

***Rawsonia lucida* Harv. & Sond.** Tree. Moist or riverine forest, 1951 m. Melly 0060 (EA).

**F19. Acanthaceae**

10 Genera, 18 species

***Acanthopale pubescens* (Lindau ex Engl.) C.B. Clarke.** Shrub. Wet Forest, 1762–1947 m. Melly 0069 (EA), Melly 0270 (EA), Bamps 6475 (EA), SAJIT 006935 (EA, HIB), SAJIT 006997 (EA, HIB).

***Acanthus eminens* C.B. Clarke**. Shrub. Upland forest, 1924 m. Melly 0101 (EA).

***Acanthus polystachius* Delile.** Perennial herb. Forest clearings, wooded grassland, 2005 m. Melly 0122 (EA).

***Acanthus pubescens* (Thoms. ex Oliv.) Engl.** Shrub. Forest edges, wet grasslands, rocky hills, 1830 m. Hill 698 (EA).

***Barleria ventricosa* Nees.** Herb. Under forests, edges and disturbed areas, 1941 m. Melly 0239 (EA).

***Brillantaisia vogeliana* (Nees) Benth.** Herb. Forest undergrowth, 2005 m. FOKP 1514 (EA, HIB).

***Brillantaisia owariensis* P. Beauv.** Herb. Wet lowland and montane forests, often along streams or in damp places, riverine or along river banks, swampy forest, 750–1850 m. Dale 3208 (EA).

***Dyschoriste radicans* Nees.** Herb. Common along roadsides, 2015 m. FOKP 1644 (EA, HIB).

***Isoglossa punctata* (Vahl) Brummitt & Wood.** Herb. Locally common in montane rain forest, 1350–2400 m. FOKP 1495 (EA).

***Justicia betonica* L.** Herb. Grassland, Wet forests, riversides, 700–2220 m. Tweedie 2985 (EA).

***Justicia pinguior* C.B. Clarke.** Herb. Common in wooded grasslands, 2149 m. FOKP 1690 (EA).

***Justicia flava* (Forssk.) Vahl.** Herb. Wooded grassland, 1951 m. Melly 0006 (EA).

***Mimulopsis arborescens* C.B. Clarke.** Tree. Upland forest, 1973 m. Cock 009 (EA).

***Mimulopsis solmsii* Schweinf.** Woody herb. Upland forest, 1986 m. Melly 0116 (EA).

***Pseuderanthemum ludovicianum* (Büttner) Lindau.** Woody herb. Riverine, forest undergrowth, 1740 m. Melly 0312 (EA).

***Thunbergia alata* Bojer ex Sims.** Herb. Forest edge, bushland, thicket, 1995 m. Melly 0134 (EA).

***Thunbergia paulitschkeana* Beck.** Herb. In forest margin, grassland of upland forest 1904–2017 m. Melly 0117 (EA), SAJIT 007014 (EA, HIB).

***Thunbergia usambarica* Lindau.** Herb. Upland forest on the forest edges, 1881–2073 m. FOKP 1654 (EA, HIB), Williams and Piers 594 (EA), SAJIT 006943 (EA, HIB), SAJIT 006983 (EA, HIB), SAJIT 006987 (EA, HIB), SAJIT 007026 (EA, HIB).

**F20. Alismataceae**

1 Genus, 1 species

***Alisma plantago-aquatica* L.** Herb. Wet places, especially by streamsides, 1884–2052 m. FOKP 1538 (EA, HIB), SAJIT 006639 (EA, HIB), SAJIT 006947 (EA, HIB).

**F21. *Amaranthaceae***

3 Genera, 4 species

***Achyranthes aspera* L.** Annual or perennial herb. Disturbed dry places, 1778 m. Melly 0269 (EA).

***Cyathula cylindrica* Moq.** Herb. Bushland, 1936 m. Melly 0097 (EA).

***Cyathula uncinulata* (Schrad.) Schinz.** Herb. Forest margins, hedges, roadside, stream banks, 2026 m. FOKP 1394 (EA), SAJIT 006662 (EA, HIB).

***Sericostachys scandens* Gilg & Lopr.** Shrub. Moist riverine, forest edge, 1733 m. Melly 0299 (EA).

**F22. Amaryllidacaee**

2 Genera, 2 species

***Crinum kirkii* Baker.** Herb. Grassland, 1900 m. Ekkens 2731 (EA).

***Scadoxus multiflorus* (Martyn) Raf.** Herb. Rocky places in forest edges, riverine forest, 2002–2052 m. FOKP 1642 (EA, HIB), SAJIT 007081 (EA, HIB).

**F23. Anacardiaceae**

1 Genus, 2 species

***Searsia longipes* (Engl.) Moffett.** Tree. Wooded grassland, 1964 m. FOKP 1574 (EA).

***Searsia pyroides* (Burch.) Moffett.** Shrub or small tree. Bushland, wooded grassland, dry forest margins, along stream banks, 2004–2058 m. FOKP 1560 (EA, HIB), FOKP 1601 (EA, HIB).

**F24. Annonaceae**

2 Genera, 2 species

***Artabotrys likimensis* De Wild.** Shrub or Liana. Evergreen rainforest, 1884–1973 m. Battiscombe 570 (EA), Melly 0055 (EA), Melly 0107 (EA), SAJIT 006678 (EA, HIB), SAJIT 006937 (EA, HIB).

***Monanthotaxis schweinfurthii* (Engl. & Diels) Verdc.** Shrub or Liana. Evergreen forest, often riverine, 1830–1966 m. Dale 3171 (EA), FOKP 1661 (EA, HIB).

**F25. Apiaceae**

1 Genus, 1 species

**Heteromorpha arborescens var. abyssinica (Hochst. ex A. Rich.) H. Wolff.** Shrub or tree. Evergreen bushland or riverine, dry forest on edges, rocky grassland, 1150–2650 m. Melly 0197 (EA).

**F26. Apocynaceae**

11 Genera, 13 species

***Carissa spinarum* L.** Shrub. Forest margins, bushland, wooded grassland, thickets, especially in rocky places, 2120 m. Melly 0202 (EA).

***Cynanchum altiscandens* K. Schum.** Climber. Upland forest edges, 2014 m. FOKP 1647 (EA).

***Funtumia africana* (Benth.) Stapf.** Tree. Moist upland forest, 1625 m. SAJIT 006680 (EA, HIB).

*Gomphocarpus physocarpus E. Meyer. Shrub. Forest edge, disturbed places, seasonal swampy grassland and roadsides, 1769–1920m. FOKP 1671 (EA, HIB), Melly 0280 (EA).

***Landolphia buchananii* (Hallier f.) Stapf.** Liana or scandent shrub. Riverine forest, relict montane evergreen forest, 1977–2143 m. Melly 0180 (EA), SAJIT 006630 (EA, HIB), FOKP 1545 (EA, HIB).

***Mondia whitei* (Hook. f.) Skeels.** Climber. Upland forest, 2026 m. FOKP 1718 (EA, HIB).

***Oncinotis tenuiloba* Stapf.** Climber. Riverine forests margins, 1888 m. Melly 0246 (EA).

***Pergularia daemia* (Forssk.) Chiov.** Twinning herb. Upland forest, 2057–2071 m. SAJIT 006629 (EA, HIB), SAJIT 007031 (EA, HIB).

***Periploca linearifolia* Quart.-Dill. & A. Rich.** Climber. Upland forest on road sides, 2010–2186 m. Melly 0127 (EA), SAJIT 006609 (EA, HIB), SAJIT 006934 (EA, HIB), FOKP 1506 (EA, HIB).

***Tabernaemontana stapfiana* Britten.** Tree. Moist disturbed forest, 1921 m. SAJIT 006934 (EA, HIB).

***Vincetoxicum anomalum* (N.E. Br.) Meve & Liede.** Climber. Upland forest, 2118–2148 m. SAJIT 006604 (EA, HIB), SAJIT 006932 (EA, HIB), Melly 0192 (EA).

***Vincetoxicum heterophylla* (A. Rich.) Vatke.** Climber. Upland forest on forest Margins, 1996 m. SAJIT 007016 (EA, HIB).

***Vincetoxicum sylvaticum* (Decne.) Kuntze.** Climber. Rare in bushland, 1920–1967 m. FOKP 1307 (EA, HIB), FOKP 1662 (EA, HIB).

**F27. Araceae**

2 Genera, 3 species

***Culcasia falcifolia* Engl.** Climber. Upland forest, 1944–2026 m. SAJIT 006636 (EA, HIB), FOKP 1381 (EA, HIB), Melly 0057 (EA).

***Culcasia scandens* P. Beauv.** Herb. Rainforest, riverine or swamp forest, 1965 m. Agnew and Musumba 8592 (EA).

***Sauromatum venosum* (Dryand. ex Aiton) Kunth.** Herb. Upland forest, uncommon in savannah, 2019–2056 m. FOKP 1631 (EA, HIB), SAJIT 007030 (EA, HIB).

**F28. Araliaceae**

5 Genera, 6 species

***Astropanax abyssinicus* (Hochst. ex A. Rich.) Seem.** Tree. Wet upland forest, 1981 m. FOKP 1384 (EA, HIB).

***Astropanax volkensii* (Harms) Lowry, G. M. Plunkett, Gostel & Frodin.** Tree. Wet or dry upland forest, 1896–1965 m. Melly 0102 (EA), FOKP 1395(EA, HIB).

***Cussonia spicata* Thunb.** Tree. Riverine, grassland forest, 2327 m. FOKP 1484 (EA, HIB).

**Heteromorpha arborescens var. abyssinica (Hochst. ex Rich.) H. Wolff.** Shrub. In montane and riverine woodland, in forest margins and in secondary regrowth, 2140 m. FOKP 1638 (EA, HIB).

***Polyscias fulva* (Hiern) Harms.** Tree. Moist upland forest, 2026–2334 m. Melly 0194 (EA), FOKP 1487 (EA, HIB).

***Schefflera myriantha* (Baker) Drake** Tree. Upland forest, 1997 m. SAJIT 007018 (EA, HIB).

**F29. Asparagaceae**

5 Genera, 11 species

***Asparagus africanus* Lam.** Shrub. Forest edges, bushy woodland, grassland, 1995 m. FOKP 1377 (EA).

***Asparagus racemosus* Willd.** Woody Climber or scrambling shrub. Forest margins, drier bushland, 1945 m. Melly 0220 (EA).

***Chlorophytum blepharophyllum* Schweinf. ex Baker.** Herb. Locally common in burnt grassland, 1828 m. Battiscombe K657 (EA).

***Chlorophytum cameronii* (Baker) Kativu.** Herb. Highland grassland, often on rocky slopes, 1706 m. Hill 1(EA).

***Chlorophytum comosum* (Thunb.) Jacques.** Herb. Shady wet places in forest, locally found in burnt grassland, 1828–1967 m. FOKP 1664 (EA, HIB), Gillett 16716 (EA).

***Chlorophytum gallabatense* Schweinf. ex Baker.** Herb. Grassland, open woodlands and shallow soils, after first rains, 1676 m. Hill 740 (EA).

***Chlorophytum nubicum* (Baker) Kativu.** Herb. Upland grasslands, 1676 m. Hill 692(EA).

***Dracaena laxissima* Engl.** Shrub. Upland forest, 1818–1991 m. Melly 0114 (EA), Melly 0260 (EA).

***Dracaena steudneri* Engl.** Shrub. Moist upland forest, 2087 m. FOKP 1777 (EA, HIB).

***Ledebouria revoluta* (L. f.) Jessop.** Herb. Upland forest, 1900–2009 m. Ekkens 2454, FOKP 1569 (EA, HIB).

***Ornithogalum gracillimum* R. E. Fr.** Herb. Grassland, 2125 m. FOKP 1616 (EA, HIB).

**F30. Asphodelaceae**

1 Genus, 1 species

***Aloe elgonica* Bullock.** Shrub. Shallow soils or in pockets on rocky slopes, 2133 m. G650 (EA), Reynold 7522 (EA).

**F31. Asteraceae**

36 Genera, 50 species

*Acanthospermum glabratum (DC.) Wild. Herb. Grassland, 1995 m. FOKP 1554 (EA, HIB).

***Acmella caulirhiza* Delile.** Herb. Swampy or seasonally wet sites, river banks, forest margins, cultivated areas, 2058 m. Melly 0155 (EA).

***Adenostemma caffrum* DC.** Herb. Wet grounds and swamps especially in disturbed sites, 1050–2300 m. Hill 151(EA), FOKP 1419 (EA, HIB).

***Ageratina adenophora* (Spreng.) R. M. King & H. Rob.** Shrub. Riverine, swampy sites, 2183 m. FOKP 1504 (EA, HIB).

*Ageratum conyzoides L. Herb. Weed in cultivation, pioneer on disturbed land, grassland, 1808 m. Melly 0027 (EA).

***Artemisia afra* Jacq. ex Willd.** Woody herb or shrub. Burnt areas, upland bushland edges, 1889 m. Melly 0244 (EA).

***Baccharoides dumicola* (S. Moore) Isawumi, El-Ghazaly & B. Nord.** Shrubby herb or small shrub. Wet grassland, mashes, riverine, 1967 m. FOKP 1669 (EA, HIB).

***Baccharoides lasiopus* (O. Hoffm.) H. Rob.** Herb. Upland forest on the edges, 1864–2058 m. FOKP 1330, Melly 0037 (EA), Melly 0157 (EA), SAJIT 006664 (EA, HIB).

***Berkheya spekeana* Oliv.** Herb. Wooded grassland, 2149 m. FOKP 1684 (EA, HIB).

***Bidens kilimandscharica* (O. Hoffm.) Sherff.** Herb. Upland forest edges, 1962 m. Melly 0002 (EA).

***Blumea axillaris* (Lam.) DC.** Herb. Local by streamsides and naturally disturbed places, 1920 m. FOKP 1323 (EA, HIB).

***Cineraria deltoidea* Sond.** Herb. Hillside, shrubland, roadsides, forest edges and cliﬀs, 1835 m. Melly 0021 (EA).

***Conyza hochstetteri* Sch.Bip. ex A. Rich.** Herb. Very common in disturbed grassland, 1987 m. FOKP 1585 (EA, HIB).

***Conyza schimperi* Sch. Bip. ex A. Rich.** Herb. Upland grassland, especially on impeded drainage, 1864 m. FOKP 1339 (EA, HIB).

***Crassocephalum montuosum* (S. Moore) Milne-Redhead.** Herb. Upland forest, valley, 1950 m. Kamau 278 (EA).

***Crassocephalum picridifolium* (DC.) S. Moore.** Herb. Swamps and river edges, 2026 m. SAJIT 006663 (EA, HIB).

***Crassocephalum rubens* (Juss. ex Jacq.) S. Moore.** Herb. Disturbed, often cultivated ground, 1677 m. 492 (EA).

***Crassocephalum vitellinum* (Benth.) S. Moore.** Herb. Upland forest, farming land, 1966 m. SAJIT 007032 (EA, HIB).

***Crepis rueppellii* Sch. Bip.** Herb. Highland grassland, 1600–3200 m. FOKP 1643 (EA, HIB).

***Dichrocephala integrifolia* (L. f.) Kuntze.** Herb. Disturbed wet habitats, 1880–1958 m. SAJIT 007037 (EA, HIB), Melly 0105 (EA).

***Distephanus biafrae* (Oliv. & Hiern) H. Rob**. Scrambling low shrub. Locally common in bushland in Western Kenya, 1040–2400 m. Dale 1012 (EA).

***Erigeron trilobus* (Decne.) Boiss.** Herb. Common in disturbed places in dry grassland, bushland, 1846–2001 m. Melly 0032 (EA), Melly 0132 (EA).

***Gamochaeta purpurea* (L.) Cabrera.** Herb. In gardens, arable cultivation, 1999 m. FOKP 1576 (EA, HIB).

***Gerbera viridifolia* (DC.) Sch. Bip.** Shrub. Upland forest, on roadsides, 1500–2100 m. Gilbert and Mesfin 6704 (EA).

***Guizotia scabra* (Vis.) Chiov.** Herb. Upland grassland, 2183 m. FOKP 1498 (EA, HIB).

***Gymnanthemum amygdalinum* (Delile) Sch. Bip. ex Walp.** Herb. Disturbed cultivated area, hillside, bushland, 1872 m. Melly 0031 (EA).

***Gymnanthemum auriculiferum* (Hiern) Isawumi.** Shrub or tree. Forest margins, riverine, lakeshores, bushland derived from disturbed upland forests, 1920 m. FOKP 1320 (EA).

***Gymnanthemum urticifolium* (A. Rich.) H. Robinson.** Shrub. Forest edges, 2026 m. Tweedie 1371 (EA, HIB).

***Helichrysum argyranthum* O. Hoffm.** Shrub. Upland forest, streamsides, 2026 m. SAJIT 006644 (EA, HIB).

***Helichrysum forskahlii* (J. F. Gmel.) Hilliard & B. L. Burtt.** Herb. Rocky grassland, 1200–3500 m. FOKP 1614 (EA).

***Helichrysum globosum* Sch. Bip.** Herb. Upland grassland, 1920 m. FOKP 1321 (EA, HIB).

***Helichrysum pedunculatum* Hilliard & B. L. Burtt.** Herb. Grassland, 1985 m. FOKP 1598 (EA, HIB).

***Kleinia abyssinica* (A. Rich.) A. Berger.** Herb. Bushland, grassland, 1849 m. FOKP 1357 (EA, HIB).

***Laggera brevipes* Oliv. & Hiern.** Herb. Forest clearings and margins, 1789 m. Melly 0278 (EA).

***Laggera elatior* R. E. Fr.** Herb. Moist upland forest, rocky places, 2148 m. SAJIT 006617 (EA, HIB).

***Lactuca glandulifera* Hook. f.** Woody herb. Moist sites along streams, forest margins, in grassland, bushland, cultivated land, 1200–3600 m. Gilbert 6883(EA).

***Linzia gerberiformis* (Oliv. & Hiern) H. Rob.** Herb. Seasonally burned grassland and wooded grassland, 1864–2149 m. Archer 247 (EA), FOKP 1333 (EA, HIB), FOKP 1686 (EA, HIB).

***Linzia ituriensis* (Muschl.) H. Rob.** Perennial herb. Wet forest margins, 2005 m. FOKP 1515 (EA, HIB).

***Lipotriche scandens* (Schumach. & Thonn.) Orchard.** Woody herb. Evergreen forest margins, river, lake and swamp margins, moist grassland, 1857–2250 m. Chandler 421 (EA), SAJIT 006965 (EA, HIB).

***Microglossa pyrifolia* (Lam.) Kuntze.** Shrub. Forest margins, waste shrubland, 1950 m. Ls 503 (EA).

***Mikania chenopodifolia* Willd.** Shrub. Marshy area, 1823 m. Melly 0046 (EA).

***Solanecio angulatus* (Vahl) C. Jeffrey.** Climber. Upland forest, 2026 m. GBK 003/057/2001 (EA), SAJIT 006642 (EA, HIB).

***Solanecio mannii* (Hook. f.) C. Jeffrey.** Shrub or tree. Dry or evergreen forest margins, secondary forest, riverine forest, on rocky slopes in bushland, 1891 m. Melly 0029 (EA).

***Senecio ruwenzoriensis* S. Moore.** Herb. Disturbed montane forests, often as boundary plant, 1892–2334 m. FOKP 1485 (EA, HIB), FOKP 1634, (EA, HIB), Melly 0125 (EA), Melly 0219 (EA).

***Sonchus camporum* (R. E. Fr.) Boulos ex C. Jeffrey.** Perennial herb. Grassland, 1800–2300 m. Dauglish 68 (EA).

***Sphaeranthus suaveolens* (Forsk.) DC.** Herb. Found near freshwater, often in dry streambeds, 1864 m. FOKP 1324 (EA, HIB).

*Tagetes minuta L. Shrub. Upland arable land, 1962–2076 m. FOKP 1658 (EA, HIB).

***Taraxacum officinale* Wiggers.** Herb. Riverine, forest edge, 1880–2076 m. Melly 0106 (EA), Melly 0165 (EA).

***Vernonia galamensis* (Cass.) Less.** Shrub. Clearings in woodland and woodland edges, 2184 m. FOKP 1502 (EA, HIB).

***Vernonia urticifolia* A. Rich.** Shrub. Upland forest on edges, 2026–2183 m. FOKP 1503 (EA, HIB), SAJIT 006650 (EA, HIB).

**F32. Balsaminaceae**

1 Genus, 7 species

***Impatiens burtonii* Hook. f.** Herb. Swampy sites of montane forests, forest edges, 2150 m. Verdcourt 1639.

***Impatiens digitata* Warb.** Herb. Forest edges, 1939 m. Melly 0005 (EA).

***Impatiens hochstetteri* Warb.** Herb. Streamsides in wet forests, 1885–2012 m. FOKP 1450 (EA, HIB), FOKP 1587 (EA, HIB), Melly 0071 (EA), Melly 0227, SAJIT 006946 (EA, HIB).

***Impatiens meruensis* Gilg.** Herb. Highland forest, marshes and streamside, 2051 m. FOKP 1539 (EA, HIB).

***Impatiens niamniamensis* Gilg.** Herb. Upland forest, 1918–2013 m. FOKP 1364 (EA, HIB), FOKP 1464 (EA, HIB), Melly 0109 (EA), SAJIT 006961 (EA, HIB).

***Impatiens pseudoviola* Gilg.** Herb. Wet places in lower highland forests, 1859 m. Melly 0256 (EA).

***Impatiens stuhlmannii* Warb.** Herb. Wet forests, 1821–2027 m. FOKP 1315 (EA, HIB), FOKP 1456 (EA, HIB), Melly 0104 (EA), Melly 0218 (EA), SAJIT 006675 (EA, HIB), SAJIT 006978 (EA, HIB).

**F33. Basellaceae**

1 Genus, 1 species

***Basella alba* L.** Climber. Hill Side, thickets, hedges, riverine vegetation (margins), cultivations, on swampy ground, 1953 m. Melly 0011 (EA).

**F34. Bignoniaceae**

2 Genera, 2 species

***Kigelia africana* (Lam.) Benth. subsp. *Africana*.** Tree. Riverine evergreen forest, forest margins, 1736–1826 m. Melly 0259 (EA), Melly 0279 (EA).

**Kigelia africana subsp. moosa (Sprague) Bidgood & Verdc.** Shrub or tree. Riverine evergreen forest, forest margins, 1864 m. FOKP 1328 (EA, HIB).

***Spathodea campanulata* P. Beauv.** Tree. Scattered tree grassland, upland forest, riverine, 2025 m. Melly 0207 (EA).

**F35. Boraginaceae**

2 Genera, 3 species

***Cynoglossum coeruleum* A. DC.** Perennial herb. Sub montane grassland, forest clearings, along the paths, 2368 m. FOKP 1489 (EA, HIB).

***Cynoglossum lanceolatum* Forssk.** Annual herb. Grassland, riverbanks, riverine forest, roadsides, cultivated ground, hillside, bushland, 1852 m. FOKP 1486 (EA, HIB), Melly 0034 (EA).

***Ehretia cymosa* Thonn.** Tree. Upland moist forest or road patches, 2008–2026 m. Melly 0143 (EA), SAJIT 006668 (EA, HIB).

**F36. Brassicaceae**

1 Genus, 1 species

***Cardamine africana* L.** Herb. Floor of highland forest and riverine, 1975–2016 m. FOKP 1369, FOKP 1526, Melly 0072, SAJIT 006951 (EA).

**F37. Cactaceae**

1 Genus, 1 species

***Rhipsalis baccifera* (J. S. Muell.) Stearn.** Epiphyte. Upland dry forest, 1758–1960 m. FOKP 1396 (EA, HIB), FOKP 1470 (EA, HIB), Melly 094 (EA), Melly 0309 (EA).

**F38. Campanulaceae**

3 Genera, 4 species

***Canarina abyssinica* Engl.** Climber. Wet forest, 1927 m. FOKP 1458 (EA, HIB).

***Canarina eminii* Asch. & Schweinf.** Climber. Wet forest, 2000 m. SAJIT 007011 (EA, HIB).

***Lobelia giberroa* Hemsl.** Shrub. Forest margins, swamps, riverine forest, 1935 m. Melly 0215 (EA).

***Wahlenbergia hookeri* (C.B. Clarke) Tuyn.** Annual herb. Grassland or woodland, often on rocky places or cultivated ground, 800–1950 m. Tallantire 124 (EA).

**F39. Cannabaceae**

4 Genera, 5 species

***Cannabis sativa* L.** Herb. Upland forest, 0–2100 m. James (EA).

***Celtis africana* Burm. f.** Tree. Riverine forest, dry evergreen forest, 2125 m. FOKP 1630 (EA, HIB).

***Celtis gomphophylla* Baker.** Tree. Forest edge, moist evergreen forest, 1200–1750 m. Melly 0123 (EA).

***Chaetachme aristata* Planch.** Shrub or Small bushy tree. Understorey, margins of uplands rainforests, 1746 m. Melly 0291 (EA).

***Trema orientalis* (L.) Blume.** Shrub or tree. Woodland forest, 2133 m. Melly 0184 (EA).

**F40. Capparaceae**

2 Genera, 2 species

***Maerua triphylla* A. Rich.** Tree. Upland forest, 2076 m. Melly 0161 (EA).

***Ritchiea albersii* Gilg.** Shrub or tree. Forest margins, forest patches, riverine forest, plantation understorey, 1980–2019 m. FOKP 1548 (EA, HIB), FOKP 1629 (EA, HIB), SAJIT 007020 (EA, HIB).

**F41. Caryophyllaceae**

1 Genus, 1 species

***Drymaria cordata* (L.) Willd. ex Roem. & Schult.** Herb. Hedges, forest margins, path sides in the wetter forest, 1920–1943 m. FOKP 1316 (EA, HIB), Melly 0084 (EA).

**F42. Celastraceae**

6 Genera, 7 species

***Gymnosporia arbutifolia* (Hochst. ex A. Rich.) Loes.** Tree. Bushland, riverine or swampy places, bushed grassland, 1989–2125 m. FOKP 1555 (EA, HIB), FOKP 1622 (EA, HIB), SAJIT 007007 (EA).

***Gymnosporia heterophylla* (Eckl. & Zeyh.) Loes.** Tree. In Forest Margin., 1985–2052 m. FOKP 1541 (EA, HIB), FOKP 1556(EA, HIB), Melly 0090 (EA), Melly 0149(EA), SAJIT 007000 (EA, HIB).

***Loeseneriella africana* (Willd.) N. Hallé.** Climbing Shrub. Riverine, also in forest or rocky woodland, 1873–2071 m. FOKP 1349 (EA, HIB), SAJIT 006621 (EA, HIB), SAJIT 006939 (EA, HIB).

***Mystroxylon aethiopicum* (Thunb.) Loes. subsp. *Aethiopicum*.** Tree. Upland forest, 2074–2140 m. FOKP 1637 (EA, HIB), Melly 0152 (EA).

***Simirestis brianii* N. Hallé.** Woody climber. Upland forest, 1936 m. Melly 0216 (EA).

***Salacia cerasifera* Wele. Ex Oliv.** Shrub. Disturbed river valley, 1773–2087 m. Dale 3424(EA), Melly 0290(EA), FOKP 1675(EA, HIB), SAJIT 006998 (EA, HIB).

***Simirestis goetzei* (Loes.) N. Hallé ex R. Wilczek.** Shrub. Woodland, forest thicket, 2019–2361 m. FOKP 1496(EA, HIB), FOKP 1595 (EA, HIB).

**F43. Colchicaceae**

1 Genus, 1 species

***Gloriosa superba* L.** Herb. Shrubland, bushland, 1836 m. Williams EAH 10576 (EA), Piers 598 (EA), Melly 0051 (EA).

**F44. Combretaceae**

1 Genus, 2 species

***Combretum paniculatum* Vent.** Vigorous Climber or scrambling shrub. Forest margins, riverine forest, wooded grassland, 1933 m. Melly 0238 (EA).

***Combretum molle* R. Br. ex G. Don.** Tree. Wooded grassland, woodland, rocky hillsides, 2125 m. FOKP 1613 (EA, HIB).

**F45. Commelinaceae**

4 Genera, 9 species

***Aneilema minutiflorum* Faden.** Herb. Montane and riverine forests, forest edge, 2078 m. Melly 0169 (EA).

***Commelina benghalensis* L.** Herb. Riverine, grassland, bushland, 1947 m. Melly 0070 (EA).

***Commelina africana* L.** Herb. Grassland, 1962 m. FOKP 1655 (EA, HIB), Siemens 101 (EA).

***Commelina aspera* G. Don ex Benth.** Herb. Upland forest, 1920 m. William 611 (EA).

***Commelina latifolia* Hochst. ex A. Rich.** Herb. Weed of cultivation and disturbed habitats, sometimes in bushland and forest edges, 1828 m. Hill 295 (EA).

***Commelina subulata* Roth.** Herb. Common in black cotton soils, 1828 m. Hill 103 (EA).

***Floscopa glomerata* (Willd. ex Schult. & Schult. f.) Hassk.** Herb. Stagnant water pools in forest, 1676–1828 m. Gilbert and Mesfin 6738 (EA), Hill 77A (EA).

***Murdannia semiteres* (Dalzell) Santapau.** Herb. Edges of temporary pools in rocky areas, 1676 m. Hill 34 (EA).

***Murdannia simplex* (Vahl) Brenan.** Herb. Swamps, grassland and rocky places, 1676 m. Hill 102 (EA).

**F46. Convolvulaceae**

3 Genera, 5 species

***Cuscuta kilimanjari* Oliv.** Climber. Common in upland forest, 2076 m. FOKP 1544 (EA, HIB), Melly 0160 (EA).

***Dichondra repens* J. R. Forst. & G. Forst.** Herb. Weed of irrigated grasslands, lawns and roadsides, 1969 m. FOKP 1435 (EA).

***Ipomoea hederifolia* L.** Annual herb. Waste places, thickets, cliffs locally established riverine forests, 0–1650 m. Meibertzhagen in E.A.H. 11364 (EA).

***Ipomoea tenuirostris* Choisy.** Climber. Upland forest on the edges, 1827–2018 m. FOKP 1308 (EA), Melly 0206 (EA), Melly 0255 (EA).

***Ipomoea wightii* (Wall.) Choisy.** Climber. Upland forest on hill Side, 1942–2071 m. Melly 0009 (EA), SAJIT 006627 (EA, HIB).

**F47. Cornaceae**

1 Genus, 1 species

***Alangium chinense* (Lour.) Harms.** Tree. Wet upland forest or semi-deciduous upland forest, 2023 m. FOKP 1518 (EA, HIB).

**F48. Crassulaceae**

2 Genus, 3 species

***Crassula granvikii* Mildbr.** Herb. Damp open soils, 1864–1927 m. FOKP 1355 (EA, HIB), FOKP 1451 (EA).

***Kalanchoe crenata* (Andrews) Haw.** Herb. Upland forest grassland, 1974–2125 m. FOKP 1611 (EA), SAJIT 007005 (EA, HIB).

***Kalanchoe prittwitzii* Engl.** Succulent herb. Upland forest thickets, bushland, 2125 m. FOKP 1605 (EA, HIB).

**F49. Cucurbitaceae**

7 Genera, 12 species

***Coccinia barteri* (Hook. f.) Keay.** Climber. Lowland forest margins, 1843 m. SAJIT 006676 (EA, HIB), SAJIT 006976 (EA, HIB).

***Coccinia subsessiliflora* Cogn.** Climber. Upland forest twining on tree trunks, 1979 m. Melly 0112 (EA), SAJIT 006676 (EA, HIB), SAJIT 006969 (EA, HIB). New record for Kenya.

***Cucumis ficifolius* A. Rich.** Climber. Upland grassland and patchsides, 1936 m. Melly 0092 (EA).

***Cucumis oreosyce* H. Schaef.** Climber. Upland grassland edges, 1873–1950 m. FOKP 1353 (EA, HIB), Melly 0233 (EA).

***Diplocyclos palmatus* (L.) C. Jeffrey**. Climber perennial. Forest edge of upland forest, 1973 m. Glasgow 46/42 (EA).

***Lagenaria abyssinica* (Hook. f.) C. Jeffrey.** Climber. Upland forest, mashy places, 1823–2026 m. FOKP 1653 (EA, HIB), Melly 0041 (EA), Melly 0242 (EA), SAJIT 006641 (EA, HIB), Melly 0214 (EA).

***Momordica cissoides* Benth.** Climber. Upland forest, 1743–1872 m. Melly 0295 (EA), SAJIT 006974 (EA, HIB).

***Momordica foetida* Schumach.** Climber. Forest edges, old cultivation and disturbed places in wet regions, 1855–2132 m. SAJIT 006667 (EA, HIB), SAJIT 006970 (EA, HIB), SAJIT 006972 (EA, HIB), Melly 0189, (EA).

***Momordica friesiorum* (Harms) C. Jeffrey.** Climber. Upland forest edges, wet bushlands, 1849 m. FOKP 1342 (EA, HIB).

***Pilogyne minutiflora* (Cogn.) W. J. de Wilde & Duyfjes.** Climber. Hillside, bushland, damp places in grassland, margins of swamps, 1867 m. Melly 0028 (EA).

***Zehneria longiflora* G. W. Hu & Q. F. Wang.** Climber. Twining on trunks and branches of trees and shrubs in upland forest, 1748–1991 m. FOKP 1352 (EA, HIB), Melly 0226 (EA), Melly 0274 (EA), SAJIT 006669 (EA, HIB), SAJIT 006670 (EA, HIB), SAJIT 006672 (EA, HIB), SAJIT 006679 (EA, HIB), SAJIT 006958 (EA, HIB), SAJIT 006959 (EA, HIB), Melly 0093 (EA), Melly 0115 (EA), Melly 0119 (EA), FOKP 1385 (EA, HIB), SAJIT 006981 (EA, HIB). New species.

***Zehneria scabra* Sond.** Perennial Climber. Forest edges, old cultivation, and bushland, 1911–2062 m. FOKP 1427 (EA, HIB), SAJIT 006973 (EA, HIB), FOKP 1527 (EA, HIB), Melly 0135 (EA), Melly 0224(EA), Melly 0231(EA), SAJIT 006665(EA, HIB), SAJIT 006966 (EA), SAJIT 006968 (EA).

**F50. Cyperaceae**

9 Genera, 26 species

***Carex chlorosaccus* C.B. Clarke.** Herb. Moist forest, damp swamps, 1650 m. Lye and Katende 4961 (EA).

***Carex lycurus* K. Schum.** Herb. Upland river-sides, 1965 m. Siemens 91 (EA).

***Courtoisina assimilis* (Steud.) Maquet.** Herb. Disturbed bare soil in wetlands, 1828 m. Hill 40 (EA).

***Cyperus ajax* C.B. Clarke.** Herb. Highland wet forest, near streams or in clearings, 1965 m. Siemens 57, Siemens 63 (EA).

***Cyperus aterrimus* Hochst. ex Steud.** Herb. Upland grassland, 1965 m. Siemens 46 (EA).

***Cyperus cyperoides* (L.) Kuntze.** Herb. Montane forest, 1676–1927 m. Maluki 1085 (EA), FOKP 1439 (EA, HIB).

***Cyperus dichrostachyus* Hochst. ex A. Rich.** Herb. Highland swamps, 1965 m. Siemens 103, FOKP 1577 (EA, HIB).

***Cyperus fischerianus* Schimp. ex A. Rich.** Herb. Moist upland forest, 1965 m. Bogdan 4260 (EA), Siemens 104 (EA).

***Cyperus latifolius* Poir.** Herb. In Forest Margin, wetland, 1975–1988 m. FOKP 1551 (EA, HIB), SAJIT 007001 (EA, HIB), SAJIT 007004 (EA, HIB).

***Cyperus marquisensis* F.Br.** Herb. Wet disturbed places, 1700 m. Hohl 102 (EA).

***Cyperus platycaulis* Baker.** Herb. Wet areas, 1965 m. Siemens 45 (EA).

***Cyperus rigidifolius* Steud.** Herb. Upland grazing fields, 1900–1965 m. FOKP 1582 (EA, HIB), Siemens 64 (EA), Hohl 94 (EA).

***Cyperus schimperianus* Steud.** Perennial Herb. Riverine, 1770 m. Melly 0315 (EA).

***Fimbristylis dichotoma* (L.) Vahl.** Herb. Riverine, 1700–1987 m. Hohl101 (EA), FOKP 1581 (EA, HIB).

***Fimbristylis subaphylla* Boeckeler.** Herb. Upland forest, 1700 m. Agnew and Musumba 8613 (EA).

***Kyllinga bulbosa* P. Beauv.** Herb. Grassland damp sites, a weed in lawns, roadsides, 1975–2032 m. FOKP 1549 (EA, HIB), Hooper and Townsend 1528, SAJIT 007002 (EA, HIB), Hill 42 (EA).

***Kyllinga eximia* C.B. Clarke.** Herb. Shallow sandy soils in bushland, 2125 m. FOKP 1610 (EA, HIB).

***Kyllinga odorata* Vahl.** Herb. Riverine, 2001 m. FOKP 1420 (EA, HIB).

***Kyllinga pulchella* Kunth.** Herb. Woodland edges, 1828 m. Siemens 65 (EA).

***Lipocarpha chinensis* (Osbeck) J. Kern.** Herb. Permanent swamps and wet places, 1828 m. Hill 288 (EA).

***Mariscus dubius* (Rottb.) Kük. ex C. E. C. Fisch. subsp. *Dubius*.** Herb. Shallow soil seepage, 1700 m. Hohl 94 (EA).

***Mariscus hemisphaericus* (Boeckeler) C.B. Clarke.** Herb. Moist grassland 2052 m. FOKP 1542 (EA).

***Mariscus longibracteatus* Cherm.** Herb. Marshy places, 1987 m. FOKP 1583 (EA, HIB), Siemens 62 (EA).

***Mariscus tomaiophyllus* (K. Schum.) C.B. Clarke.** Perennial herb. Montane swamps, 1800–2900 m. Siemens 50 (EA).

***Pycreus elegantulus* (Steud.) C.B. Clarke.** Herb. Disturbed upland marshes, 1986 m. FOKP 1579 (EA, HIB).

***Pycreus flavescens* (L.) P. Beauv. ex Rchb.** Herb. Soggy open places in the rainy season, often on black cotton soils, 2038 m. SAJIT 007023 (EA, HIB).

***Scleria bulbifera* Hochst. ex A. Rich.** Herb. Seasonally wet grassland, 2125 m. Dyer 1180 (EA).

**F51. Dichapetalaceae**

1 Genus, 1 species

***Dichapetalum madagascariense* Poir.** Liana, shrub or small tree. Regenerating forest, moist upland forest, 1967 m. FOKP 1668 (EA, HIB).

**F52. Dioscoreaceae**

1 Genus, 4 species

***Dioscorea asteriscus* Burhill.** Climber. Upland forest, 1866 m. SAJIT 006980 (EA, HIB).

***Dioscorea praehensilis* Benth.** Climber. Rainforest, riverine, 1700–1782 m. Bally 13687 (EA), Melly 0297 (EA, HIB).

***Dioscorea schimperiana* Hochst. ex Kunth.** Climber. Wooded grassland, 2118–2148 m. Melly 0191 (EA), Williams 577 (EA), SAJIT 006619 (EA, HIB).

***Dioscorea quartiniana* A. Rich.** Climber. Woody grassland and bushland, 1923 m. Melly 0087 (EA).

**F53. Ebenaceae**

1 Genus, 1 species

***Diospyros abyssinica* (Hiern) F. White.** Tree. Riverine forests, 1930 m. Melly 0089 (EA).

**F54. Euphorbiaceae**

10 Genera, 14 species

***Acalypha villicaulis* Hochst. ex A. Rich.** Herb or weak shrub. Hill Side, Highland forest, warm areas, 1950 m. Melly 0013 (EA).

***Acalypha volkensii* Pax.** Shrub. Upland grassland, 1983 m. FOKP 1546 (EA, HIB).

***Alchornea hirtella* Benth.** Shrub or tree. Moist forests, 1974 m. FOKP 1389 (EA, HIB).

***Alchornea laxiflora* (Benth.) Pax & K. Hoffm.** Shrub or tree. Moist or dry forests, 1858 m. SAJIT 006975 (EA).

***Croton macrostachyus* Hochst. ex Delile.** Tree. Forest margins, 1945–2108 m. FOKP 1624 (EA, HIB), Melly 0008 (EA).

***Erythrococca trichogyne* (Műll. Arg.) Prain.** Shrub. Moist forests and riverine, 1849–1967 m. FOKP 1344 (EA, HIB), Melly 0062 (EA), Melly 0234 (EA), SAJIT 006992 (EA, HIB).

***Erythrococca bongensis* Pax.** Tree. Dry forest, riverine bushland, woodland, shrubby grassland, Wooded bushland, or thicket, 1892–1910 m. SAJIT 006991 (EA, HIB), SAJIT 006393 (EA, HIB).

***Erythrococca fischeri* Pax.** Shrub. Grassland, woodland, 1973–2086 m. Melly 0064 (EA), Melly 0171 (EA), SAJIT 006626 (EA, HIB).

**Macaranga capensis var. kilimandscharica (Pax) Friis & M. G. Gilbert.** Tree. Moist upland forests, often in forest edges, 1970 m. Melly 0066 (EA).

***Neoboutonia macrocalyx* Pax.** Tree. Upland forests, mostly on edges and in clearings, 1934–2134 m. Melly 0091 (EA), Makin 16706, Melly 0195 (EA).

***Ricinus communis* L.** Woody perennial herb. Bushland, Path sides, 1931 m. Melly 0085 (EA).

***Shirakiopsis elliptica* (Hochst.) Esser.** Tree. Riverine forests, moist and dry forest mostly near water, 150–2148 m. Brunt1328 (EA), SAJIT 006612 (EA, HIB).

***Tragia brevipes* Pax.** Herb. Dry upland forest, 1854–1967 m. FOKP 1667 (EA, HIB), Melly 0035 (EA).

***Tragiella natalensis* (Sond.) Pax & K. Hoffm.** Twining or scrambling perennial herb. Upland forest on roadsides, Forest margins, 1961–2087 m. FOKP 1663 (EA, HIB), FOKP 1673 (EA, HIB), SAJIT 007033 (EA, HIB).

**F55. Fabaceae**

21 Genera, 31 species

***Acacia lahai* Steud. & Hochst. ex Benth.** Tree. Forms dense woodland where upland forest has disappeared, or invading grassland, 2023 m. FOKP 1589 (EA, HIB).

***Acacia pentagona* (Schum. & Thonn.) Hook. f.** Robust liana. In evergreen forest, 1707 m. Melly 0287 (EA).

***Albizia amara* (Roxb.) B. Boivin.** Tree. Bushland, wooded grassland, 1957 m. Melly 0004 (EA).

***Alysicarpus glumaceus* (Vahl) DC.** Herb. Common in seasonally flooded grassland, especially short grass on shallow soils, 2149 m. FOKP 1513 (EA, HIB).

***Amphicarpaea africana* (Hook. f.) Harms.** Climber. Upland forest edges, 2071 m. SAJIT 006624 (EA, HIB).

*Caesalpinia decapetala (Roth) Alston. Tree. Forest edge, 1969 m. Melly 0003 (EA).

***Calpurnia aurea* (Aiton) Benth.** Shrub. Moist forest margins and riverine forest, 1995–2114 m. FOKP 1564 (EA, HIB), FOKP 1623 (EA, HIB), SAJIT 006652 (EA, HIB).

***Chamaecrista kirkii* (Oliv.) Standl.** Herb. Grassland, marshy places, forest margins and clearings, 2125 m. FOKP 1617 (EA, HIB).

***Chamaecrista mimosoides* (L.) Greene.** Herb. Common in disturbed places and open habitats on shallow soils, 2133 m. Melly 0185 (EA).

***Crotalaria agatiflora* Schweinf.** Perennial herb or shrub. Bushed grassland, roadsides, forest margins, waste places, 1888 m. SAJIT 006989 (EA, HIB).

***Crotalaria axillaris* Aiton.** Shrub. Bushland, 1987–2052 m. FOKP 1537 (EA, HIB), FOKP 1547 (EA, HIB).

***Crotalaria mauensis* Baker f.** Shrub. Under forest, 2073 m. Melly 0172 (EA).

***Dalbergia lactea* Vatke.** Shrub. Riverine, 1856–2026 m. Melly 0232 (EA), FOKP 1312 (EA, HIB), SAJIT 006985 (EA, HIB), Melly 0126, (EA).

***Desmodium adscendens* (Sw.) DC.** Annual, perennial herb. Shaded forest clearings or margins, old cultivated fields, grassland of stream banks, 2135 m. FOKP 1513 (EA, HIB), Melly 0178 (EA).

***Desmodium repandum* (Vahl) DC.** Herb. Upland forest, riverine by path sides, 1938 m. Melly 0235 (EA).

***Dumasia villosa* DC.** Climber. Wet and evergreen forest, thickets, 1974 m. FOKP 1390 (EA, HIB).

***Eriosema buchananii* Baker f.** Shrubby herb. Upland forest grassland, 1821–2148 m. Melly 0050 (EA), SAJIT 006615(EA, HIB).

***Eriosema psoraloides* (Lam.) G. Don.** Perennial herb or subshrub. Wooded grassland, shrub, bushland, forest margins, 1861 m. Melly 0190 (EA), Melly 0254 (EA).

***Haydonia triphylla* R. Wilczek.** Herb. Grassland, 1500–2100 m. Whyte.

***Indigofera homblei* Baker f. & Martin.** Shrub. Upland forest, 2134 m. Melly 0187 (EA).

***Lablab purpureus* (L.) Sweet.** Climbing herb. Riverine forests, marshy area, 1818 m. Melly 0038 (EA).

***Neonotonia wightii* (Wight & Arn.) J. A. Lackey.** Climber. Forest edges, disturbed ground, 2071 m. SAJIT 006622 (EA, HIB).

*Senna didymobotrya (Fresen.) H. S. Irwin & Barneby. Shrub. Bushland, 1939 m. Melly 0095 (EA).

*Senna obtusifolia (L.) H. S. Irwin & Barneby. Shrub. Riverine, hillside, shrubland, 1896 m. Melly 0026 (EA).

*Senna septemtrionalis (Viv.) H. S. Irwin & Barneby. Shrub. Upland forest, riverine, grassland, waste places, 1992–2076 m. FOKP 1565 (EA, HIB), Melly 0159 (EA).

***Sesbania sesban* (L.) Merr.** Soft wooded tree. Riverine, occur in dense stands, 1877 m. Melly 0249 (EA).

***Trifolium burchellianum* Ser.** Herb. Moist upland grassland, forest, 1929 m. FOKP 1472 (EA, HIB).

***Trifolium usambarense* Taub.** Annual or perennial herb. Marshy places, 1800 m. Melly 0265 (EA).

***Vigna luteola* (Jacq.) Benth.** Herb. Swampy grassland and forest margins, 1920 m. FOKP 1672 (EA, HIB).

***Vigna parkeri* Baker.** Perennial herb. Grassland, thicket, forest margins, weed in cultivation, 1785–1927 m. FOKP 1452 (EA, HIB), Melly 0313 (EA).

***Zornia setosa* Baker f.** Herb. Grassland with impeded drainage, 2014–2018 m. FOKP 1645 (EA, HIB), FOKP 1646 (EA, HIB), Melly 0213 (EA).

**F56. Gentianaceae**

1 Genus, 1 species

***Anthocleista grandiflora* Gilg.** Tree. Riverine, swampy places in forests, 1883 m. SAJIT 006949 (EA, HIB).

**F57. Hamamelidaceae**

1 Genus, 1 species

***Trichocladus ellipticus* Eckl. & Zeyh.** Shrub or tree. Dry or moist forest, 1764 m. Melly 0310 (EA).

**F58. Hydrocharitaceae**

3 Genera, 3 species

***Egeria densa* Planch.** Aquatic free-flowing herb. Water pools, 1828 m. Hill 690 (EA).

***Ottelia ovalifolia* Rich.** Aquatic perennial with submerged or floating leaves. Locally common in pools of water, 1864 m. FOKP 1329 (EA).

***Vallisneria spiralis* L.** Submerged herb. Recorded in the Nandi hills on water pools, 2025 m. Odero 1E (EA).

**F59. Hypericaceae**

2 Genera, 2 species

***Hypericum revolutum* Vahl.** Shrub. Grows along streams in upland forest thickets, dry bushland, 1760 m. Melly 0308 (EA).

***Harungana madagascariensis* Lam. Ex Poir.** Tree. Moist forests margins, 1757 m. Melly 0311 (EA).

**F60. Hypoxidaceae**

1 Genus, 3 species

***Hypoxis angustifolia* Lam.** Herb. Grassland in upland forest, 1730–2149 m. FOKP 1559 (EA, HIB), FOKP 1689 (EA, HIB), Davide 7121 (EA).

***Hypoxis obtusa* Burch. ex Ker Gawl.** Herb. Common in burnt grassland and shallow soils, 2005 m. Hill 91 (EA).

***Hypoxis villosa* L. f.** Herb. Burnt grassland on shallow or rocky soils, 1962 m. FOKP 1659 (EA, HIB).

**F61. Icacinaceae**

1 Genus, 1 species

***Apodytes dimidiata* E. Mey. ex Arn.** Shrub or tree. Upland rain forest, and forest patches, 1751–2000 m. FOKP 1572 (EA, HIB), Melly 0281 (EA).

**F62. Iridaceae**

2 Genera, 2 species

***Aristea alata* Baker.** Rhizomatous perennial herb. Forest margins and grass glades, 2195 m. Mainwring 10559 (EA).

***Freesia laxa* (Thunb.) Goldblatt & J. C. Manning.** Herb. Rocky grassland, 1828 m. Hill 2 (EA).

**F63. Lamiaceae**

14 Genera, 28 species

***Achyrospermum parviflorum* S. Moore.** Herb. Undergrowth of rain-forest, swamp forest riverine forest, 1050–1700 m. Tweedie 4150 (EA).

***Clerodendrum formicarum*** Gűrke. Supported shrub. Upland forest, 1872–1952 m. Melly 0056 (EA), Melly 0252 (EA).

***Clerodendrum johnstonii* Oliv.** Shrub. Upland forest and thicket, or disturbed scrubland, marshy area, 1823–2148 m. Melly 0047 (EA), SAJIT 006601 (EA, HIB).

***Clerodendrum melanocrater*** Gürke. Climber. Upland forest, 1973 m. SAJIT 006671 (EA, HIB).

***Clerodendrum rotundifolium* Oliv.** Shrub. Wooded grassland, riverine woodland, 1946 m. Melly 0059 (EA).

***Clinopodium abyssinicum* (Hochst. ex Benth.) Kuntze.** Herb. Woodland, 1864 m. FOKP 1325 (EA, HIB).

***Fuerstia africana* T.C.E. Fr.** Herb. Grassland and understorey of upland forest, 2023–2143 m. FOKP 1590 (EA, HIB), Melly 0163 (EA)

***Isodon ramosissimus* (Hook. f.) Codd.** Herb. Forest undergrowth and margins upland grasslands, descending altitudes along rivers, 750–2100 m. Tweedie 4153 (EA).

***Leonotis martinicensis* (Jacq.) J. C. Manning & Goldblatt.** Herb. Open ground, disturbed places especially in farmland, 2058 m. Melly 0156 (EA).

***Leonotis ocymifolia* (Burm. f.) Iwarsson.** Shrub. Upland forest, 2121 m. Melly 0188 (EA).

***Leucas deflexa* Hook. f.** Herb. Upland grassland, 1967 m. FOKP 1666 (EA, HIB).

***Ocimum gratissimum* L.** Perennial herb or subshrub. Disturbed bushland, Hillside, shrubland, 1905 m. Melly 0022 (EA).

***Ocimum lamiifolium* Hochst. ex Benth.** Shrub. Upland forest, 1930–2258 m. FOKP 1479 (EA, HIB), SAJIT 006999 (EA, HIB).

***Ocimum obovatum* E. Mey. ex Benth.** Herb or shrub. Upland grassland, on poor drainage, 2149 m. FOKP 1682 (EA, HIB).

***Orthosiphon rubicundus* (D. Don) Benth.** Herb. Common in wooded grassland, 2036 m. FOKP 1507 (EA, HIB).

***Orthosiphon schimperi* Benth.** Herb. Wooded grassland, 1927 m. FOKP 1460 (EA, HIB).

***Platostoma africanum* P. Beauv.** Perennial herb. Damp places in grassland, roadside, by streams, forest edges, shrubland, 1857–1920 m. FOKP 1310 (EA, HIB), Melly 0023 (EA).

***Platostoma denticulatum* Robyns.** Herb. Riverside, wetland often in partial shade at forest edges, roadside grassland, 1883 m. SAJIT 006948 (EA, HIB).

***Plectranthus alpinus* (Vatke) Ryding.** Shrub. Shady forest, 1963–1991 m. FOKP 1413, Melly 0236 (EA).

***Plectranthus barbatus* Andrews.** Shrub. Bushland, rocky grassland, 1823 m. Melly 0044 (EA).

***Plectranthus bojeri* (Benth.) Hedge.** Herb. Hillside, shrubland, on shallow soils, 1804 m. Melly 0018 (EA).

***Plectranthus kamerunensis*** Gürke. Shrub. Upland forest, 2010 m. FOKP 1406 (EA, HIB).

***Plectranthus mollis* (Aiton) Spreng.** Shrub. Upland forest, 1904 m. FOKP 1367 (EA, HIB), Melly 0103 (EA).

***Plectranthus punctatus* (L. f.) L’Hér.** Herb. Disturbed upland forests, 2001 m. FOKP 1422 (EA, HIB).

***Plectranthus sylvestris*** Gürke. Herb or shrub. Disturbed places of upland forest, plantation of exotics, 1943 m. SAJIT 007034 (EA, HIB).

***Pycnostachys niamniamensis*** Gürke. Annual herb. Forest undergrowth and margins upland grasslands, descending altitudes along rivers, 750–2100 m. Duke 3411 (EA).

***Rotheca myricoides* (Hochst.) Steane & Mabb.** Shrub. Upland dry forest, grazed bushland, grassland, 1861–2134 m. FOKP 1641 (EA, HIB), Melly 0036 (EA), Melly 0053 (EA), Melly 0201 (EA).

***Tetradenia riparia* (Hochst.) Codd.** Shrub. On the rocky slopes, 2148 m. SAJIT 006613 (EA, HIB).

**F64. Linderniaceae**

1 Genus, 3 species

***Craterostigma newtonii* (Engl.) Eb. Fisch., Schäferh. & al.** Herb. Local on shallow soils, 2120–2125 m. FOKP 1604 (EA, HIB), Melly 0203 (EA).

***Craterostigma nummulariifolium* (D. Don) Eb. Fisch., Schäferh. & Kai Müll.** Herb. Weed of cultivation, streamside in both open country and wet, mossy, evergreen forests and in thickets on boulder-strewn hillsides, 1942 m. Rodgers 714 (EA), Melly 0082 (EA).

***Craterostigma pumilum* Hochst.** Herb. Over rocks, in montane forest, 2149 m. FOKP 1685 (EA, HIB).

**F65. Loranthaceae**

4 Genera, 6 species

***Agelanthus pennatulus* (Sprague) Polhill & Wiens.** Epiphyte. In Forest Margin, 1988 m. SAJIT 007009 (EA, HIB). Vulnerable.

***Agelanthus platyphyllus* (Hochst. ex A. Rich.) Balle.** Shrub. Deciduous woodland and woodland grassland, almost always on Combretum or Terminalia, 1200–1600 m. Davidse 7125 (EA).

***Agelanthus zizyphifolius* (Engl.) Polhill & Wiens.** Epiphytic Shrub. Under forest, 2026 m. SAJIT 006653 (EA, HIB).

***Englerina woodfordioides* (Schweinf.) Balle.** Shrub. Upland forest, 1998–2148 m. FOKP 1311 (EA, HIB), FOKP 1594 (EA, HIB), Melly 0130 (EA), SAJIT 006610 (EA, HIB), SAJIT 006657 (EA, HIB), SAJIT 006666, (EA, HIB).

***Phragmanthera usuiensis* (Oliv.) M. G. Gilbert.** Shrub. Upland forest, 2133 m. FOKP 1481 (EA, HIB), Melly 0186 (EA).

***Tapinanthus constrictiflorus* (Engl.) Danser.** Epiphyte. Evergreen forests, on various hosts including plantation crops, 2134–2300 m. Archer 237 (EA), Melly 0196 (EA).

**F66. Lythraceae**

1 Genus, 1 species

***Rotala tenella* (Guill. & Perr.) Hiern.** Herb. Grassland, roadside, 2002–2026 m. Melly 0137 (EA), SAJIT 006640 (EA, HIB).

**F67. Malvaceae**

8 Genera, 14 species

***Abutilon longicuspe* Hochst. ex A. Rich.** Shrub. Upland forest, forest margins, scrubland, 2026 m. SAJIT 006649 (EA, HIB).

***Dombeya burgessiae* Gerrard ex Harv.** Shrub or tree. Semi-evergreen bushland on rocky sites, riverine, wooded grassland, 2058 m. FOKP 1600 (EA, HIB).

***Dombeya torrida* (J. F. Gmel.) B. Bamps.** Tree. Upland forest, 1993 m. Melly 0139 (EA).

***Hibiscus calyphyllus* Cav.** Shrub. Rainforest, riverine forest, thickets, grassland, 2000 m. Melly 0148 (EA).

***Hibiscus macranthus* Hochst. ex A. Rich.** Small shrub. Common in forest edges and grassland in cleared forest, 2019 m. FOKP 1596 (EA, HIB).

***Kosteletzkya adoensis* (Hochst. Ex A. Rich) Mast.** A perennial herb or Shrub. Forest edges and cleared forests, 1769 m. Melly 0271 (EA).

***Malvastrum coromandelianum* (L.) Garcke.** Woody annual or perennial. Wasteland, bushland edges and grassland, 2022 m. Melly 0205 (EA).

***Pavonia urens* Cav.** Shrub. Road patches in upland forest, 1758–2026 m. FOKP 1652 (EA, HIB), Melly 0142 (EA), Melly 0263 (EA), SAJIT 006638, (EA, HIB).

***Pavonia procumbens* (Wight) Walp.** Woody herb. Dry woodland and rocks, 2014 m. FOKP 1650 (EA, HIB).

***Sida acuta* Burn. f.** Herb. Disturbed places on grassland forest, 1993 m. Melly 0118 (EA).

***Sida rhombifolia* L.** Herb. Hillside, grassland, bushland, 1889 m. Melly 0030 (EA).

***Triumfetta brachyceras* K. Schum.** Woody herb or shrub. Forest edges and along roadsides, 1751–1920 m. FOKP 1670 (EA, HIB), Melly 0272 (EA).

***Triumfetta rhomboidea* Jacq.** Shrub. Weed of cultivation, hillside, shrubland, 1890 m. Melly 0020 (EA).

***Triumfetta tomentosa* Bojer.** Shrub. Forest clearings, old fields, often secondary vegetation, 2009 m. Melly 0146 (EA).

**F68. Melastomataceae**

3 Genera, 3 species

***Dissotis speciosa* Taub.** Woody herb or shrub. Marshy places, valley grassland, 1784 m. Melly 0301 (EA).

***Dupineta brazza* (Cogn.) Veranso-Libalah & G. Kadereit.** Herb. Forest margins, valley and upland grassland and deciduous bushland, 900–1800 m. Williams 571 (EA).

***Tristemma mauritianum* J. F. Gmel.** Shrub or herb. Swampy places, 1800 m. Melly 0302 (EA).

**F69. Meliaceae**

3 Genera, 3 species

***Lepidotrichilia volkensii* (Gürke) Leroy.** Tree. Forest margins, 1929–1981 m. FOKP 1386 (EA, HIB), Melly 0098 (EA).

***Trichilia emetica* Vahl.** Tree. Riverine, riverine forest and woodland, lake-shore thicket, 1945 m. SAJIT 007035 (EA, HIB).

***Turraea holstii*** Gürke. Tree. Upland forest, 1915–2026 m. FOKP 1335 (EA, HIB), SAJIT 006647 (EA, HIB), SAJIT 006938 (EA, HIB).

**F70. Melianthaceae**

1 Genus, 1 species

***Bersama abyssinica* Fresen.** Tree. Upland forest, 1805–2392 m. FOKP 1474 (EA, HIB), Melly 0081 (EA), Melly 0237 (EA), Melly 0276 (EA), SAJIT 006602 (EA, HIB), SAJIT 007012 (EA, HIB).

**F71. Menispermaceae**

3 Genera, 6 species

***Cissampelos friesiorum* Diels.** Climber. Upland forest on edges, 2012 m. SAJIT 007015 (EA, HIB).

***Cissampelos mucronata* A. Rich.** Climber. Riverine, 2148 m. SAJIT 006599 (EA, HIB).

***Cissampelos pareira* L.** Climber. Upland forest, woodland, 1934–2023 m. FOKP 1591 (EA, HIB), Melly 0077 (EA).

***Stephania abyssinica* (Quart. -Dill. & A. Rich.) Walp.** Climber. Upland forest, 2026 m. SAJIT 006654 (EA, HIB), SAJIT 006659 (EA, HIB).

***Stephania cyanantha* Welw. ex Hiern.** Climber. Hill Side of upland forest, 1739–2026 m. FOKP 1455 (EA, HIB), FOKP 1571 (EA, HIB), Melly 0025 (HIB), Melly 0296 (HIB), SAJIT 006651 (EA, HIB).

***Tiliacora funifera* (Miers) Oliv.** Woody Liana. Rainforest, riverine forest, moist shady places in woodland, 1744–1973 m. Melly 0294 (EA), SAJIT 006673 (EA, HIB).

**F72. Monimiaceae**

1 Genus, 1 species

***Xymalos monospora* (Harv.) Baill.** Tree. Moist upland forest, 1849–2005 m. FOKP 1356 (EA, HIB), FOKP 1512 (EA, HIB).

**F73. Moraceae**

4 Genera, 11 species

***Dorstenia barnimiana* Schweinf.** Herb. Wet upland forest, 2015 m. FOKP 1570 (EA, HIB).

***Ficus asperifolia* Miq.** Shrub. Forest margins and thickets, 1887 m. Melly 0245 (EA).

***Ficus cyathistipula* Warb.** Tree. Rain-forest and other wetter evergreen forest, 700–1800 m. Green 12 (EA).

***Ficus exasperata* Vahl.** Tree. Wet forests on the margins, 1–1850 m. Lucas 146 (EA).

***Ficus lutea* Vahl.** Tree. Rain-forest and other wetter evergreen forest, riverine and ground-water forests sometimes on rocks, 1931 m. FOKP 1441 (EA, HIB).

***Ficus ottoniifolia* (Miq.) Miq.** Tree. Upland forest on the margins, woodland forest, riverine, 1757–2070 m. FOKP 1678 (EA, HIB), Melly 0063 (EA), Melly 0300 (EA), SAJIT 006971 (EA, HIB), SAJIT 006990 (EA, HIB).

***Ficus sur* Forssk.** Tree. Riverine forest, bushland, groundwater forest, 1–2100 m. Dale 3405.

***Ficus sycomorus* L.** Tree. Forest, woodland and wooded grassland, sometimes along rivers and lakes or amongst rocks, planted for ornament and back-cloth, 1772–2076 m. Williams 578 (EA), Melly 0168 (EA), Melly 0267 (EA).

***Ficus thonningii* Blume.** Tree. Rain-forest and other wetter evergreen forest, riverine and groundwater forests, 1800 m. Wye 1929 (EA).

***Morus mesozygia* Stapf.** Tree. Moist forest, 1200–1650 m. Wormald 25 (EA).

***Trilepisium madagascariense* DC.** Tree. Rain-forest and other wetter evergreen forest, riverine and groundwater forests, 1800 m. Wye 1843(EA).

**F74. Musaceae**

1 Genus, 1 species

***Ensete ventricosum* (Welw.) Cheesman.** Giant herb. Upland forest, 1856 m. Melly 0258 (EA).

**F75. Myrtaceae**

3 Genera, 3 species

*Eucalyptus saligna Sm. Tree. Common plantation timber, 1952 m. Melly 0240 (EA).

***Psidium guajava* L.** Tree. Upland forest, bushland, 1929 m. Melly 0086 (EA).

***Syzygium guineense* (Willd.) DC.** Tree. Wooded grassland, riverine, 1746–2071 m. FOKP 1628 (EA, HIB), Melly 0173 (EA), Melly 0282 (EA).

**F76. Ochnaceae**

1 Genus, 2 species

***Ochna holstii* Engl.** Tree. In Forest, dominant trees, very big, 1985–2052 m. FOKP 1531 (EA, HIB), FOKP 1597 (EA, HIB), SAJIT 007019 (EA, HIB).

***Ochna insculpta* Sleumer.** Tree. Upland forest, woodland, 1991–2051 m. FOKP 1415 (EA, HIB), FOKP 1543 (EA, HIB), FOKP 1568, (EA, HIB).

**F77. Oleaceae**

4 Genera, 6 Species

***Chionanthus mildbraedii* (Gilg & G. Schellenb.) Stearn.** Tree. Wet upland Forest, 1831 m. Melly 0262 (EA).

***Jasminum abyssinicum* Hochst. ex DC.** Shrub. Upland evergreen bushland, 1791–1879 m. Melly 0266 (EA), SAJIT 006953 (EA, HIB).

**Jasminum grandiflorum subsp. floribundum (R. Br. ex Fresen.) P. S. Green.** Shrub or scrambler. Upland evergreen bushland, 2100 m. Hill 312 (EA).

***Olea capensis* L.** Tree. Wet and dry evergreen mixed forest, 1150–2550 m. Battiscombe 135 (EA).

***Olea welwitschii* (Knobl.) Gilg & Schellenb.** Tree. Lowland rain to upland dry evergreen forest, 750–1950 m. Battiscombe 660 (EA).

***Schrebera alata* (Hochst.) Welw.** Tree. Dry forest edges, evergreen bushland, 2143 m. Melly 0183 (EA).

**F78. Onagraceae**

1 Genus, 2 species

***Ludwigia abyssinica* A. Rich.** Succulent herb. Swampy places, 1798 m. Melly 0314 (EA).

**Ludwigia adscendens subsp. diffusa (Forssk.) P. H. Raven.** Herb. Marshy area, riverine, 1823 m. Melly 0042 (EA).

**F79. Ophioglossaceae**

1 Genus, 1 species

***Ophioglossum reticulatum* L.** Herb. In Forest margins and damp pockets of soils on the rocky outcrops, 2003 m. SAJIT 007010 (EA, HIB).

**F80. Orobanchaceae**

1 Genus, 1 species

***Orobanche minor* Sm.** Herb. Upland forest, 1991 m. FOKP 1414 (EA, HIB).

**F81. Orchidaceae**

17 Genera, 36 species

***Aerangis ugandensis* Summerh.** Herb. In moist forest, near rivers on large tree trunks and often with its roots in a deep growth of moss, 1500–2000 m. William and Piers 601 (EA).

***Angraecum erectum* Summerh.** Epiphyte. Dry forest, 1975 m. FOKP 1360 (EA, HIB).

***Angraecum humile* Summerh.** Dwarf epiphytic herb. Upper branches of forest trees, 1785 m. Melly 0268 (EA).

***Bolusiella maudiae* (Bolus) Schltr.** Epiphyte. Humid forest, 2026 m. FOKP 1680 (EA, HIB) Tweedie 230 (EA), Williams and Piers 615 (EA).

***Bulbophyllum cochleatum* Lindl.** Epiphyte. Shady forest, 2138 m. FOKP 1680 (EA, HIB).

**Bulbophyllum cochleatum var. bequaertii (De Wild.) J. J. Verm.** Epiphytic Herb. Epiphytic in rain forest, 900–2000 m. Tweedie 233 (EA).

**Bulbophyllum bidenticulatum subsp. joyceae J. J. Verm.** Epiphyte. Upland forest, 1914 m. Melly 0111 (EA).

***Bulbophyllum encephalodes* Summerh.** Epiphyte. Upland forest, 1991 m. Melly 0113 (EA).

***Bulbophyllum josephi* (Kuntze) Summerh.** Epiphyte. Upland forest, 1914 m. Melly 0110 (EA).

***Bulbophyllum intertextum* Lindl.** Epiphyte. Warm forests, 1700–2019 m. FOKP 1632 (EA, HIB).

***Cribbia brachyceras* (Summerh.) Senghas.** Epiphyte. On mossy trunks of trees and rocks in forests, 1500–2200 m. Tweedie 287 (EA).

***Cyrtorchis arcuata* (Lindl.) Schltr.** Epiphyte. On trees and rocks in bush, woodland and forest, 0–2000 m. FOKP 1580 (EA, HIB), William and Piers 609 (EA).

***Diaphananthe sarcophylla* (Schltr. ex Prain) P. J. Cribb & Carlsward.** Herb. Dry upland forest on trees, 1964 m. FOKP 1575 (EA, HIB).

***Disperis anthoceros* Rchb.f.** Herb. Under forest on ground litter in evergreen forest, 2082 m. Melly 0153 (EA).

***Disperis dicerochila* Summerh.** Herb. Upland forest, leaf litter, mossy branches and on rocks in upland rain forest, 1886 m. Melly 0108 (EA).

***Epipogium roseum* (D. Don) Lindl.** Herb. Upland forest on leaf mould in wet forests, 2030–2033 m. FOKP 1477 (EA, HIB), FOKP 1517 (EA, HIB), FOKP 1625 (EA, HIB), FOKP 1649 (EA, HIB), SAJIT 007021 (EA, HIB).

***Eulophia galeoloides* Kraenzl.** Herb. Upland forest on trees, 2023–2055 m. FOKP 1593 (EA, HIB), SAJIT 007029 (EA, HIB).

***Eulophia horsfallii* (Bateman) Summerh.** Herb. Robust Orchid of swamps and river edges, 1925 m. FOKP 1465 (EA, HIB).

***Eulophia latilabris* Summerh.** Herb. Grassland and swampy places, 1812 m. Melly 0264 (EA).

***Eulophia stachyodes* Rchb. f.** Herb. Grassland, bushland and woodland, 950–2100 m. Rodgers 719 (EA).

***Eulophia streptopetala* Lindl.** Herb terrestrial. In grassland, bushland and woodland, 950–2100 m. SAJIT 006660 (EA, HIB).

***Habenaria chirensis* Rchb. f.** Herb. Damp grassland, swamps, wet places amongst rocks, 2026 m. SAJIT 006655 (EA, HIB).

***Kylicanthe rohrii* (Rchb. f.) Descourvières & Farminhão.** Epiphyte. Dry upland forest, 2070 m. FOKP 1679 (EA, HIB).

***Microcoelia globulosa* (Ridl.) L. Jonss.** Epiphytic herb. Riverine forests, 1740–1966 m. FOKP 1660 (EA, HIB), Melly 0285 (EA).

***Nervilia bicarinata* (Blume) Schltr.** Herb. Upland forest, 1975 m. FOKP 1392 (EA, HIB).

***Nervilia kotschyi* (Rchb. f.) Schltr.** Herb. Short grassland and amongst bushes and shrubs, 1864–2020 m. FOKP 1337 (EA, HIB) FOKP 1626 (EA, HIB), SAJIT 007024 (EA, HIB).

***Nervilia lilacea* Jum. & H. Perrier.** Herb. Rain forest floor margins, 2010–2052 m. FOKP 1530 (EA, HIB), FOKP 1567 (EA, HIB). New record for Kenya.

***Polystachya adansoniae* Rchb. f.** Epiphytic Herb. Dry forest, 1961–1976 m. FOKP 1400 (EA, HIB), Melly 0221 (EA).

***Polystachya bennettiana* Rchb. f.** Epiphyte. In dry forest, on big trees, 2034–2125 m. FOKP 1620, SAJIT 007028 (EA, HIB).

***Polystachya cultriformis* (Thouars) Lindl. ex Spreng.** Epiphyte. Riverine, 1985–2125 m. FOKP 1444 (EA, HIB), FOKP 1621 (EA, HIB), SAJIT 007017, (EA, HIB).

***Polystachya fusiformis* (Thouars) Lindl.** Epiphytic herb. Upland forest, 2062 m. FOKP 1528 (EA, HIB).

***Polystachya golungensis* Rchb. f.** Epiphytic herb. Light shade on trees or rocks, 2052 m. FOKP 1532 (EA, HIB).

***Polystachya tenuissima* Kraenzl.** Epiphyte. Upland forest, 1927–2092 m. FOKP 1447 (EA, HIB), FOKP 1633 (EA, HIB), SAJIT 007025 (EA, HIB).

***Polystachya simplex* Rendle.** Epiphyte. Dry highland forest, 2125 m. FOKP 1501 (EA, HIB), SAJIT 006656 (EA, HIB).

***Polystachya spathella* Kraenzl.** Epiphyte. Highland forest, 2040 m. SAJIT 007027 (EA, HIB).

***Rhipidoglossum rutilum* (Rchb. f.) Schltr.** Epiphyte. Evergreen forest, 1930–1939 m. FOKP 1448 (EA, HIB), Melly 0088 (EA), Melly 0222 (EA

***Satyrium crassicaule* Rendle.** Herb. Wetland, swamps, 1851–1964 m. FOKP 1446 (EA, HIB), SAJIT 006986 (EA, HIB), SAJIT 007038 (EA, HIB).

**F82. Oxalidaceae**

1 Genus, 2 species

***Oxalis corniculata* L.** Herb. Grassland, Disturbed ground, 2076 m. Melly 0167 (EA).

***Oxalis obliquifolia* Steud. ex A. Rich.** Herb. Grassland, shallow soils, 830–3300 m. Tulin 81 (EA).

**F83. Passifloraceae**

2 Genera, 4 species

***Adenia bequaertii* Robyns & Lawalrée.** Climber. Forest edge, 1996 m. Melly 0136 (EA).

***Adenia cissampeloides* (Planch. ex Hook.) Harms.** Climber. Dry or moist forest on margins, 1849–2148 m. Melly 0170 (EA), FOKP 1345 (EA, HIB), Melly 0225 (EA), SAJIT 006600 (EA, HIB), SAJIT 006931 (EA, HIB), SAJIT 006933 (EA, HIB).

**Adenia lobata subsp. rumicifolia (Engl. & Harms) Lye.** Woody Climber. Moist evergreen (riverine) forest, 1757–1989 m. Melly 0292 (EA), Gillet 16701 (EA), SAJIT 007006 (EA, HIB).

*Passiflora edulis Sims. Climber. Cultivated, forest edges and disturbed places, 1719 m. Melly 0273 (EA).

**F84. Penaeaceae**

1 Genus, 1 species

***Olinia rochetiana* A. Juss.** Shrub or tree. Drier upland forest, found also in forest remnants such as fire-induced thickets, 2071 m. SAJIT 006631 (EA, HIB).

**F85. Peraceae**

1 Genus,1 species

***Clutia abyssinica* Jaub. & Spach.** Shrub. Secondary bushland, 2005–2326 m. FOKP 1483 (EA, HIB), FOKP 1602 (EA, HIB), Melly 0128 (EA).

**F86. Phyllanthaceae**

2 Genus, 6 species

***Bridelia micrantha* (Hochst.) Baill.** Shrub or tree. Usually riverine or in forest margins, Less often in bushed or wooded grassland, 1751 m. Melly 0288 (EA).

***Phyllanthus boehmii* Pax.** Herb. Grassland, 1987 m. FOKP 1584 (EA, HIB).

***Phyllanthus fischeri* Pax.** Shrub. Woodland, Forest edge and clearings, river line bushes, 1450–2700 m. FOKP 1561 (EA, HIB).

***Phyllanthus maderaspatensis* L.** Sub Shrub. Grassland in dry upland forest, 2076 m. Melly 0162 (EA).

***Phyllanthus nummulariifolius* Poir.** Shrub. Dry scattered tree grassland, 2125 m. FOKP 1615 (EA, HIB).

***Phyllanthus ovalifolius* Forssk.** Shrub or tree. Upland forest, bushland, 1745 m. Melly 0283 (EA).

**F87. Phytolaccaceae**

1 Genus, 2 species

***Phytolacca dodecandra* L’Hér.** Climbing Shrub. Bushland and cleared forest, 1960 m. Melly 0228 (EA).

*Phytolacca octandra L. Shrub. Moist forest margin, 2151 m. Melly 0193 (EA).

**F88. Piperaceae**

2 Genera, 5 species

***Peperomia abyssinica* Miq.** Succulent perennial herb. Wet upland forest, 1973 m. Melly 0316 (EA).

***Peperomia fernandopoiana* C. DC.** Herb. Highland forest on trees, 1872–1973 m. SAJIT 006954 (EA, HIB).

***Peperomia retusa* (L. f.) A. Dietr.** Epiphytic herb. Upland forest, 1889–2026 m. Melly 0068 (EA), SAJIT 006648 (EA, HIB), SAJIT 006960 (EA, HIB).

***Peperomia tetraphylla* (G. Forst.) Hook. & Arn.** Epiphytic creeping herb. Wet upland forests, occasionally in dry savannah edges. Forms mats on horizontal branches, 1936–2016 m. FOKP 1525 (EA, HIB), Melly 0223 (EA).

***Piper capense* L. f.** Shrubby herb or climber. Wet highland forest, valley, 1969 m. Melly 0017 (EA).

**F89. Pittosporaceae**

1 Genus, 1 species

***Pittosporum viridiflorum* Sims.** Tree or shrub. Evergreen riverine, wooded grassland, 2120–2148 m. FOKP 1619 (EA, HIB), Melly 0204 (EA), SAJIT 006618 (EA, HIB).

**F90. Plantaginaceae**

1 Genus, 2 species

***Veronica abyssinica* Fresen.** Herb. Upland grassland, 1990 m. FOKP 1578 (EA, HIB).

***Veronica javanica* Blume.** Herb. Disturbed upland grassland, sometimes weed of garden or crops, 2186 m. FOKP 1497 (EA, HIB).

**F91. Poaceae**

19 Genera, 30 species

***Adenochloa hymeniochila* (Nees) Zuloaga.** Herb. Swampy places, 1965 m. Siemens 97 (EA).

***Andropogon schirensis* Hochst.** Herb. Grassland, 1785 m. Dale 24 (EA).

***Brachiaria umbratilis* Napper.** Herb. Upland forest margins, 1676 m. Maluki 1092 (EA).

***Cenchrus clandestinus* (Hochst. ex Chiov.) Morrone.** Herb. Grassland, 1965 m. Siemens 76 (EA).

***Cenchrus macrourus* (Trin.) Morrone.** Herb. Stream banks, 1765 m. Melly 0303 (EA).

***Cenchrus unisetus* (Nees) Morrone.** Herb. Upland grassland, 1676 m. Maluki 1077 (EA).

***Chloris pycnothrix* Trin.** Herb. Very common on trampled weedy path sides, 0–2300 m. Siemens 77 (EA).

***Cynodon dactylon* (L.) Pers.** Herb. Disturbed short grassland, 0–2000 m. Siemens 72 (EA).

***Digitaria abyssinica* (Hochst. ex A. Rich.) Stapf.** Herb. Frequent weed along the road and disturbed damp grassland, 0–3000 m. Hohl 93 (EA).

***Digitaria ternata* (A. Rich.) Stapf.** Herb. In Forest Margin, wetland, 1975 m. SAJIT 007003 (EA, HIB).

***Digitaria velutina* (Forssk.) P. Beauv.** Herb. Very common in disturbed sites, 0–2300 m. Siemens 99 (EA).

***Eragrostis atrovirens* (Desf.) Trin. ex Steud.** Herb. Uncommon in wet grassland, 1667 m. Maluki 1070 (EA).

***Eragrostis exasperata* Peter.** Herb. Seasonally wet and shallow soils, 300–2000 m. Hohl 89 (EA).

***Eragrostis paniciformis* (A. Braun) Steud.** Herb. Common after rains in upland grassland in openings, 300–3000 m. Dale 1(EA).

***Eragrostis patula* (Kunth) Steud.** Herb. Common weed of dry bushland, 0–2800 m. Conell G643(EA), Hohl 103(EA).

***Eragrostis racemosa* (Thunb.) Steud.** Herb. Common in most dry upland grassland, drainage line, 1300–2600 m. Williams 613 (EA).

***Exotheca abyssinica* (Hochst. ex A. Rich.) Andersson.** Herb. Upland grassland, 1676–1826 m. Maluki 1069 (EA), Conell G640 (EA).

***Hyparrhenia cymbaria* (L.) Stapf.** Herb. Upland forest, 1828 m. Hill 82 (EA).

***Hyparrhenia filipendula* (Hochst.) Stapf.** Herb. Disturbed tall grassland, 1872 m. Dyer 1154 (EA).

***Hyperthelia dissoluta* (Nees ex Steud.) Clayton.** Herb. Upland grassland, 2133 m. Conell G642 (EA).

***Leersia hexandra* Sw.** Perennial grass. Moist, marshy areas, 1698 m. Melly 0286 (EA), Siemens 98 (EA).

***Loudetia kagerensis* (K. Schum.) C. E. Hubb. ex Hutch.** Herb. Stony soils, 1962 m. FOKP 1657 (EA, HIB), Conell G645(EA).

***Oplismenus hirtellus* (L.) P. Beauv.** Herb. Forest shade, 1965 m. Siemens 109 (EA).

***Panicum subalbidum* Kunth.** Herb. Riverine, 1965 m. Siemens 88A (EA).

***Pseudechinolaena polystachya* (Kunth) Stapf.** Herb. Seasonal swamps and along the rivers, 2011 m. FOKP 1405 (EA, HIB).

***Rhytachne rottboellioides* Desv.** Herb. In swamps and seasonally wet grassland, 1–2100 m. Brunt 1358 (EA).

***Setaria sphacelata* (Schumach.) Stapf & C. E. Hubb. ex M. B. Moos.** Herb. Upland grassland, stony hillside, 1965–3300 m. FOKP 1656 (EA, HIB), Malaki 1068 (EA), Guy 2928 (EA).

***Urochloa brizantha* (A. Rich.) R. D. Webster.** Herb. Upland grassland, 1676–1700 m. Maluki 1090 (EA), Hohl 90 (EA).

***Urochloa jubata* (Fig. & De Not.) Sosef.** Herb. Damp upland pasture, 1676 m. Maluki 1084 (EA).

***Urochloa semiundulata* (Hochst. ex A. Rich.) Ashalantha & V. J. Nair.** Herb. Disturbed bushland and grassland, 1750 m. Dale 19 (EA).

**F92. Polygonaceae**

2 Genera, 6 species

***Persicaria decipiens* (R. Br.) K. L. Wilson.** Herb. Waterside grassland, 1993 m. Melly 0140 (EA).

***Persicaria lapathifolia* (L.) Delarbre.** Herb. Marshy area, 1823 m. Melly 0048 (EA).

***Persicaria nepalensis* (Meisn.) H. Gross.** Shrub. Wet forest, 2007 m. FOKP 1423 (EA, HIB).

***Persicaria senegalensis* (Meisn.) Soják.** Herb. Common in water sites and marshes, 1927 m. FOKP 1459 (EA, HIB).

***Persicaria setosula* (A. Rich.) K. L. Wilson.** Herb. Upland forest on marshy places, 1925–1982 m. FOKP 1466 (EA, HIB), FOKP 1550 (EA, HIB), SAJIT 007039 (EA, HIB).

***Polygonum afromontanum* Greenway.** Herb. Roadside, 1942 m. Melly 0083 (EA).

**F93. Polygalaceae**

2 Genera, 3 species

***Polygala sparsiflora* Oliv.** Herb. Brachystegia woodland, bushland and grassland, sometimes in marshes and seepage areas, 1000–2200 m. Dale 3182 (EA).

***Polygala sphenoptera* Fresen.** Herb. Upland grassland, disturbed soil, 1987–2140 m. FOKP 1640 (EA, HIB), SAJIT 007008 (EA, HIB).

***Securidaca welwitschii* Oliver.** Scandent shrub or liana. Upland Evergreen Forest, 1774–1947 m. FOKP 1343 (EA, HIB), Gillett 16730 (EA), Melly 0289 (EA), SAJIT 006955 (EA, HIB), SAJIT 006984 (EA, HIB).

**F94. Primulaceae**

2 Genera, 2 species

***Embelia schimperi* Vatke.** Shrub or small tree. Upland forest, 1740–2026 m. FOKP 1558 (EA, HIB), Melly 0212 (EA), Melly 0284 (EA), SAJIT 006633 (EA, HIB), SAJIT 006950, (EA, HIB).

***Maesa lanceolata* Forssk.** Tree. Widespread secondary forest, forest margin, 1950–1997 m. Melly 0014 (EA), Melly 0145 (EA).

**F95. Proteacae**

1 Genus, 1 Species

***Protea madiensis* Oliv.** Shrub. Wooded grassland, 2149 m. FOKP 1683 (EA, HIB).

**F96. Putranjivaceae**

1 Genus, 1 species

***Drypetes gerrardii* Hutch.** Shrub. Roadside, 2000 m. Melly 0131 (EA).

**F97. Ranunculaceae**

3 Genera, 3 species

***Clematis brachiata* Thunb.** Climber. Rocky place, wooded grassland, 2148 m. SAJIT 006614 (EA, HIB).

***Ranunculus multifidus* Forssk**. Perennial herb. Marshy area, 1823 m. Melly 0049 (EA).

***Thalictrum rhynchocarpum* Quart. -Dill. & A. Rich. ex A. Rich.** Herb. Upland forest, 1946 m. Melly 0061 (EA).

**F98. Rhamnaceae**

3 Genera, 3 species

***Gouania longispicata* Engl.** Liana or sprawling shrub. Riverine forest, upland

forest (margins, clearings), 1973 m. SAJIT 006677 (EA, HIB).

***Helinus mystacinus* (Aiton) E. Mey. ex Steud.** Shrub. Upland forest, 2125 m. FOKP 1603 (EA, HIB).

***Scutia myrtina* (Burm. f.) Kurz.** Shrub. Moist, dry forest, riverine, 1947 m. Melly 0067 (EA).

**F99. Rhizophoraceae**

1 Genus, 2 species

***Cassipourea malosana* (Baker) Alston.** Tree. Bushland, understorey of moist forest, 1920 m. FOKP 1317 (EA, HIB).

***Cassipourea ruwensorensis* (Engl.) Alston.** Tree. Evergreen forest, 1952 m. Melly 0079 (EA).

**F100. Rosaceae**

3 Genera, 5 species

***Alchemilla kiwuensis* Engl.** Herb. Highland grassland or as a weed, 1981 m. FOKP 1552 (EA, HIB), Fries 613 (EA).

***Prunus africana* (Hook. f.) Kalkman.** Tree. Forest edge and grazing land, 1970 m. Moon 765(EA). Vulnerable.

***Rubus apetalus* Poir.** Shrub. Upland bushland, 1974 m. Melly 0120 (EA).

***Rubus pinnatus* Wild.** Shrub. Upland rain forest, 1823–2135 m. Melly 0040 (EA), Melly 0176 (EA).

***Rubus steudneri* Schweinf.** Shrub. Upland forest, roadside, 1993 m. Melly 0141 (EA).

**F101. Rubiaceae**

18 Genera, 23 species

***Canthium bugoyense* (K. Krause) Lantz.** Shrub. Upland forest on the margins, 1925–1951 m. Melly 0058 (EA), SAJIT 006930 (EA, HIB).

***Canthium oligocarpum* Hiern.** Shrub or tree. Moist forest, 2000 m. FOKP 1482 (EA, HIB).

***Coffea eugenioides* S. Moore.** Shrub. Dry bushland, 1955–2087 m. FOKP 1674 (EA, HIB), Gillett 16698 (EA), SAJIT 007036 (EA, HIB).

***Galium aparinoides* Forssk.** Climber. Upland forest edges, 2016 m. FOKP 1523 (EA, HIB).

***Gardenia volkensii* K. Schum.** Shrub. Riverine woodland, wooded grassland, 1–1750 m. Rindsay 1958 (EA).

***Heinsenia diervilleoides* K. Schum.** Tree. Upland forest, evergreen, rainforest, moist evergreen forest, 1815–1973 m. FOKP 1336 (EA, HIB), Melly 0248 (EA), SAJIT 006674 (EA, HIB), Tweedie 41631 (EA), SAJIT 006977 (EA, HIB).

***Hymenodictyon floribundum* (Hochst. & Steud.) B. L. Rob.** Tree. Upland Forest, 2143–2148 m. Dale 3177 (EA), Melly 0182 (EA), SAJIT 006608(EA, HIB).

***Keetia gueinzii* (Sond.) Bridson.** Climber. Moist forest, bushland, 2134–2148 m. Melly 0200 (EA), SAJIT 006603 (EA, HIB).

***Mussaenda arcuata* Lam. ex Poir.** Shrub, scandent shrub or climber. Grassland, bushland, open or closed forest, evergreen rainforest, 700–1830 m. Makin 307 (EA).

***Oldenlandia corymbosa* L.** Herb. Dry areas in upland forest, 2023 m. FOKP 1592 (EA, HIB).

***Oldenlandia herbacea* (L.) Roxb.** Herb. On rocky places, grassland, bushland, thickets, 0–2160 m. SAJIT 006607 (EA, HIB).

***Oxyanthus speciosus* DC.** Tree. Forest riverine, 1809–1873 m. FOKP 1354 (EA, HIB), Melly 0250 (EA), Melly 0306 (EA).

***Pavetta abyssinica* Fresen.** Tree. Shrubland, riverine and secondary bushland, 1836–1864 m. FOKP 1334 (EA, HIB), Melly 0052 (EA), SAJIT 006964 (EA, HIB).

***Pavetta crassipes* K. Schum.** Shrub. Wooded grassland, 2016–2071 m. Melly 0211 (EA), SAJIT 006628 (EA, HIB).

***Pentas longiflora* Oliv.** Shrubby Herb. Grassland, bushland, scrub, thicket and forest edges, sometimes in damp places usually on volcanic soils, 2148–2450 m. Puruglose 3628 (EA), SAJIT 006606 (EA, HIB).

**Psychotria capensis var. puberula (E.M.A. Petit) Verdc.** Tree. Swampy places, riverine, 1763–2026 m. FOKP 1588 (EA, HIB), Melly 0217 (EA), Melly 0304 (EA), SAJIT 006632 (EA, HIB).

***Rutidea orientalis* Bridson.** Shrub. Woodland, 1864–2026 m. FOKP 1331(EA, HIB), Melly 0080 (EA), SAJIT 006634 (EA, HIB).

***Rytigynia acuminatissima* (K. Schum.) Robyns.** Shrub. Drier forest margins, 1957–1992 m. FOKP 1382 (EA, HIB), FOKP 1566 (EA, HIB).

***Spermacoce princeae* (K. Schum.) Verdc.** Herb. Wet forests and on the forest edges, 1823–2076 m. Melly 0045 (EA), Melly 0166 (EA).

***Tarenna pavettoides* (Harv.) Sim.** Small tree or shrub. Upland forest, riverine forest or secondary bushland near forest, 0–1920 m. Girbert and Tadessa 6699 (EA), Melly 0151 (EA).

***Vangueria apiculata* K. Schum.** Tree. Thickets, riverine, wooded grassland, bushland, 1913–2000 m. Melly 0100 (EA), Melly 0121 (EA).

***Vangueria madagascariensis* J. F. Gmel.** Tree. Riverine, wooded grassland, bushland, 1861 m. SAJIT 006996 (EA, HIB).

***Vangueria volkensii* K. Schum.** Tree. Riverine, bushland, 1920–2260 m. FOKP 1309 (EA, HIB), FOKP 1480 (EA, HIB), Melly 0177 (EA).

**F102. Rutaceae**

4 Genera, 4 species

***Clausena anisata* (Willd.) Hook. f. ex Benth.** Shrub. Forest, secondary bushland, 1799 m. Melly 0275 (EA).

***Fagaropsis hildebrandtii* (Engl.) Milne-Redh.** Shrub or tree. Rocky evergreen bush or woodland or dry forest, 2046 m. FOKP 1599 (EA, HIB).

***Toddalia asiatica* (L.) Lam.** Liana. Forest margin, grassland thickets, 1998 m. Melly 0144 (EA).

***Zanthoxylum gilletii* (De Wild.) P. G. Waterman.** Tree. Rain forest, 900–2400 m. Board 5 (EA).

**F103. Salicaceae**

5 Genera, 8 species

***Casearia battiscombei* R. E. Fr.** Tree. Upland moist forest, 2278 m. FOKP 1478 (EA, HIB).

***Casearia gladiiformis* Mast.** Shrub or tree. Usually riverine or in forest margins. Less often in bushed or wooded grassland, 2078 m. Melly 0175 (EA).

***Dovyalis abyssinica* (A. Rich.) Warb.** Tree. Upland moist forest, 1928 m. FOKP 1469 (EA, HIB).

***Dovyalis macrocalyx* (Oliv.) Warb.** Shrub. Upland forest, grassland, 1867–2052 m. FOKP 1372 (EA, HIB), FOKP 1395 (EA, HIB), FOKP 1534 (EA, HIB), SAJIT 006637 (EA, HIB), SAJIT 006937 (EA, HIB).

***Flacourtia indica* (Burm. f.) Merr.** Tree or Shrub. Drier forest edges, wooded grassland or woodland, clump bushland, 2020–2138 m. FOKP 1573 (EA, HIB), Melly 0198 (EA).

***Oncoba routledgei* Sprague.** Tree or Shrub. Moist upland forest often riverine, 1859–1884 m. SAJIT 006952 (EA, HIB), SAJIT 006963 (EA, HIB).

***Oncoba spinosa* Forssk.** Tree or Shrub. Riverine forest or bushland, 1934 m. Melly 0076 (EA).

***Trimeria grandifolia* (Hochst.) Warb.** Tree. Bushland, 2013–2078 m. FOKP 1535 (EA, HIB), FOKP 1557 (EA, HIB), Melly 0174 (EA), SAJIT 006625 (EA, HIB).

**F104. Santalaceae**

1 Genus, 1 species

***Viscum schimperi* Engl.** Herb. Dry evergreen forest and associated bushland, 2179 m. FOKP 1500 (EA, HIB).

**F105. Sapindaceae**

3 Genera, 5 species

***Allophylus africanus* P. Beauv.** Shrub or tree. Riverine of grassland, termite mounds, wooded and semi-open grasslands, thickets and scrub, 30–2400 m. Battiscombe 289 (EA).

***Allophylus ferrugineus* Taub.** Tree or Shrub. Moist or dry forest, riverine, 1828 m. Siemens 6457 (EA).

***Allophylus rubifolius* (Hochst. ex A. Rich.) Engl.** Tree or Shrub. Dry bushland, woodland, moist forest, 1952–1968 m. Melly 0016 (EA), Melly 0078 (EA).

**Allophylus rubifolius var. alnifolius (Baker) Friis & Vollesen.** Shrub. Dry forest, 1869 m. Melly 0251 (EA).

***Deinbollia kilimandscharica* Taub.** Tree. Upland forest, 1828–1973 m. Melly 0065 (EA), Melly 0257 (EA), Tweedie 4158 (EA).

***Paullinia pinnata* L.** Shrubby Climber. Forest margins, gallery forest, moist thickets and scrub, 0–1600 m. Gillett 16699 (EA).

**F106. Sapotaceae**

1 Genus, 1 species

***Synsepalum cerasiferum* (Welw.) T. D. Penn.** Tree. Lowland rain-forest, groundwater forest and riverine forest, 300–1500 m. Shantz 125 (EA).

**F107. Schrophulariaceae**

1 Genera, 1 species

***Buddleja polystachya* Fresen.** Tree. Upland forest, Bushland, 1913 m. Melly 0241 (EA).

**F108. Smilacaceae**

1 Genus, 2 species

***Smilax anceps* Wild.** Climber. Wet forest, 2026–2134 m. Melly 0199 (EA), SAJIT 006658 (EA, HIB).

***Smilax aspera* L.** Climber. Evergreen upland forest, 1920 m. FOKP 1314 (EA, HIB).

**F109. Solanaceae**

4 Genera, 11 species

*Cestrum aurantiacum Lindl. Shrub. Upland forest, cultivated and disturbed ground, 2074–2183 m. FOKP 1499 (EA, HIB), Melly 0150 (EA).

***Discopodium penninervium* Hochst.** Shrub. Upland forest, 2071 m. SAJIT 006620 (EA, HIB).

*Physalis peruviana L. Herb. Cultivated fields, 1873 m. FOKP 1350 (EA, HIB).

***Solanum aculeastrum* Dunal.** Shrub. Forest margin, 1823–2026 m. Melly 0124 (EA), SAJIT 006661 (EA, HIB).

***Solanum americanum* Mill.** Shrub. Cultivated areas, 1823–2086 m. Melly 0043 (EA), Melly 0158 (EA).

***Solanum anguivi* Lam.** Shrub. Hill Side of upland forest, cultivated areas, 1863–2026 m. FOKP 1373 (EA, HIB), Melly 010 (EA), Melly 0253 (EA), Melly 0099 (EA), SAJIT 006643 (EA, HIB), SAJIT 006646 (EA, HIB).

***Solanum incanum* L.** Shrub. Waste places, secondary vegetation, a weed in grassland, 2076 m. Melly 0164 (EA).

***Solanum mauense* Bitter.** Shrub. Secondary forest, 1815 m. Melly 0019 (EA).

*Solanum mauritianum Scop. Shrub. Hillside/bushland, cultivation, 1846–2026 m. Melly 0033 (EA), SAJIT 006635 (EA, HIB).

***Solanum sessiliflorum* M. F. Dun.** Shrub. Cultivation or disturbed places, 2052 m. FOKP 1536 (EA, HIB).

***Solanum terminale* Forssk.** Shrub or Liana. Forest margins, riverine, evergreen bushlands woodland, 1927–1960 m. FOKP 1457 (EA, HIB), Melly 0012, Melly 0154 (EA), Melly 0229 (EA).

**F110. Stilbaceae**

1 Genus, 1 species

***Nuxia congesta* R. Br. ex Fresen.** Tree. Upland forest, grassland, 2148 m. SAJIT 006616 (EA, HIB).

**F111. Strombosiaceae**

1 Genera, 1 Species

***Strombosia scheffleri* Engl.** Tree. Dominant in moist forest, 1849 m. FOKP 1346 (EA, HIB).

**F112. Urticaceae**

8 Genera, 8 species

***Didymodoxa caffra* (Thunb.) Friis & Wilmot-Dear.** Herb. Disturbed places in upland forest, in shade amongst rocks, 2383 m. FOKP 1475 (EA, HIB).

***Droguetia debilis* Rendle.** Herb. Marshy areas, upland forest, 1968–2026 m. Melly 0074 (EA), SAJIT 006645 (EA, HIB).

***Elatostema monticola* Hook. f.** Herb. Riverine, wet places, 1975 m. Melly 0073 (EA).

***Laportea alatipes* Hook. f.** Herb. Disturbed places in wet upland forest, 1851–2005 m. FOKP 1418 (EA, HIB), SAJIT 006982 (EA, HIB).

***Pilea rivularis* Wedd.** Herb. Upland forest, streams, and paths, 2007 m. FOKP 1425 (EA, HIB).

***Pouzolzia parasitica* (Forssk.) Schweinf.** Herb. Upland forest, 2014 m. FOKP 1651 (EA, HIB).

***Urera hypselodendron* (Hochst. ex A. Rich.) Wedd.** Climber-woody. Moist upland forest, 2022–2071 m. SAJIT 006623 (EA, HIB), SAJIT 007013 (EA, HIB).

***Urtica massaica* Mildbr.** Herb. Upland forest, 1969–2083 m. FOKP 1436 (EA, HIB), Melly 0208 (EA).

**F113. Verbenaceae**

1 Genera, 2 species

*Lantana camara L. Shrub. Hillside, shrubland, 1802 m. Melly 0024 (EA).

*Lantana trifolia L. Shrub. Marshy area, bushland, 1823 m. Melly 0039 (EA).

**F114. Violaceae**

2 Genera, 2 species

***Afrohybanthus enneaspermus* (L.) Flicker.** Herb. Forest edge, disturbed dry bushland, 1992 m. Melly 0133 (EA).

***Rinorea brachypetala* (Turcz.) O. Ktze.** Shrub or small tree. Evergreen forests, along rivers, 850–1900 m. Gillett 16730 (EA).

**F115. Vitaceae**

4 Genera, 9 species

***Cayratia gracilis* (Guill and Perr.) Suess.** Climber. Forest edges of riverine forest, lowland forest, 1946 m. Melly 0054 (EA).

***Cissus humbertii* Robyns & Lawalrée.** Climber. Bushland, forest edges, 1700–1925 m. Melly 0096 (EA). Vulnerable.

***Cissus oliveri* (Engl.) Gilg.** Climber. Upland forest, 1960 m. FOKP 1383 (EA, HIB).

***Cyphostemma bambuseti* (Gilg & M. Brandt) Desc. ex Wild & R. B. Drumm.** Climber. Forest edge of dry upland forest, 1882–1968 m. Melly 0015 (EA), Melly 0230 (EA), Melly 0243 (EA), SAJIT 006945 (EA, HIB).

***Cyphostemma cyphopetalum* (Fresen.) Desc. ex Wild & R. B. Drumm.** Climber. Roadside, evergreen upland forest, 2001 m. Melly 0129 (EA).

***Cyphostemma kilimandscharicum* (Gilg) Desc. ex Wild & R. B. Drumm.** Climber. Upland rainforest, 1826–2052 m. FOKP 1529 (EA, HIB), Melly 0261 (EA), SAJIT 006994 (EA, HIB).

***Cyphostemma ukerewense* (Gilg) Desc.** Climber. In Forest, on tree, 1890–1916 m. SAJIT 006936 (EA, HIB), SAJIT 006944 (EA, HIB).

***Rhoicissus revoilii* Planch.** Climber. Disturbed bushland and woodland, 2143 m. Melly 0181 (EA).

***Rhoicissus tridentata* (L. f.) Wild & R. B. Drumm.** Shrub. Upland wooded grassland and forest thickets, 1992–2125 m. Melly 0138 (EA), FOKP 1612 (EA, HIB).

**F116. Xyridaceae**

1 Genus, 1 species

***Xyris straminea* L. A. Nilsson.** Herb. Edges of pools in dry places, 2050 m. Gilbert and Mesfin 6737 (EA).

**F117. Zingiberaceae**

1 Genus, 2 species

***Aframomum angustifolium* (Sonn.) K. Schum.** Herb. Riverine forest, 1912 m. SAJIT 006995 (EA, HIB).

***Aframomum zambesiacum* (Baker) K. Schum.** Herb. Along path sides in wet forest, 1903 m. SAJIT 006957 (EA, HIB).

**F118. Zygophyllaceae**

1 Genus, 1 species

***Tribulus terrestris* L.** Herb. Forest edge, 2035 m. Melly 0209 (EA).

### Discussion

The present checklist provides a comprehensive inventory of the vascular plants found in North and South Nandi forests. Our list almost doubles the previous floristic account by Girma et al. (2015) where 321 plant species in 92 families and 243 genera were recorded.

Most of the species in our checklist are present in the adjacent Kakamega rainforest (Fischer et al. 2010). This could be because in the early 20th century, Kakamega, North and South Nandi forests were once joined, forming a u-shaped forest block (Kigomo 1987; Mitchell et al. 2006; Lung and Schaab 2010). That forest block is currently divided into three different areas, namely Kakamega, North Nandi and South Nandi forests. Nandi forests play an essential role in the endeavour of conserving the Kakamega forest and they are environmentally significant as they protect the catchment of Nandi escarpment and the Lake Victoria basin.

South Nandi and North Nandi forests contain highland elements in its fauna and flora and are thus unique (Girma et al. 2015). Moreover, together with Kakamega forest, the North Nandi forest and South Nandi forest form part of the western rainforest region and the easternmost fragment of the Guinea-Congolian phytogeographical region (Mbuvi et al. 2015). The high number of threatened species in these forests indicates their importance as a global biodiversity resource (MEWNR 2015).

These forests are surrounded by many people who depend on them entirely or partially for their ecosystem services like timber, firewood, pasture, charcoal, medicinal plants for people and livestock and building materials, amongst many other functions (Fashing and Mwangi 2004; Akwee et al. 2010; Maua et al. 2018b).

Currently, this region is amongst the most densely populated rural areas in Kenya (Mitchell et al. 2006). The demand pressure on the limited resources of North and South Nandi forests is high (Kogo et al. 2019). Therefore, efforts to manage and conserve the forest’s resources in a sustainable way will be crucial to the survival of this vulnerable ecosystem (Bennun and Njoroge 1999; Matiru 2002).

## Conclusion

The description of one new species and two new records for Kenya from Nandi forests, together with this comprehensive checklist, is a clear indication that opportunities for scientific studies are abundant in North Nandi and South Nandi Forests and are of crucial importance to the conservation of these unique ecosystems. Other than this checklist, very little is known about the specifics of the community or ecosystem ecology of North and South Nandi Forests. Hence, more studies should be done in this area to fill the existing knowledge gaps.
